# System‐Level Assessment of LNG Cold Energy Utilization for Functional Expansion of Regasification Terminals

**DOI:** 10.1002/gch2.70166

**Published:** 2026-10-27

**Authors:** Jiyoung Lee, Byeongchan Ahn, Hyuntae Sohn, Yeonsoo Kim, Kiho Park, Wangyun Won

**Affiliations:** ^1^ Department of Chemical and Biological Engineering Korea University Seongbuk‐gu Seoul Republic of Korea; ^2^ Center for Hydrogen and Fuel Cell Research Korea Institute of Science and Technology (KIST) Seongbuk‐gu Seoul Republic of Korea; ^3^ Department of Chemical Engineering Kwangwoon University Nowon‐gu Seoul Republic of Korea; ^4^ Department of Chemical Engineering Hanyang University Seongdong‐gu Seoul Republic of Korea

**Keywords:** cryogenic energy recovery, energy hub, environmental techno‐economic assessment, life cycle assessment, LNG import terminal, LNG regasification, organic rankine cycle (ORC)

## Abstract

Liquefied natural gas (LNG) regasification releases substantial cryogenic energy, presenting an opportunity to reframe LNG terminals from simple gas suppliers to integrated energy hubs. This study evaluates the system‐level integration of two parallel organic Rankine cycles (ORCs) with hydrogen production pathways—proton exchange membrane water electrolysis (PEMWE) and seawater direct electrolysis (SWDE)—to assess their economic and environmental feasibility. A comprehensive techno‐economic analysis and life cycle assessment reveal that, owing to the dominant influence of LNG feedstock costs and large LNG throughput, the integration of these systems results in only marginal variations in the minimum selling price and global warming potential of natural gas. Among the evaluated pathways, ORC–PEMWE demonstrates a more favorable balance, enabling low‐carbon hydrogen production (0.42 kg CO_2_‐eq/kg‐H_2_), whereas SWDE remains constrained under the current assumptions due primarily to the conservative replacement interval and associated replacement costs, together with its higher assumed capital requirement. Furthermore, an environmental techno‐economic assessment indicates that capital expenditure‐oriented policy support is more effective than carbon credits in improving system competitiveness. Overall, this work provides a realistic, policy‐relevant framework for transforming existing LNG infrastructure into multifunctional energy systems while maintaining economic feasibility.

## Graphical abstract

Liquefied natural gas regasification terminals are reimagined as multifunctional energy hubs by recovering cryogenic energy through parallel organic Rankine cycles (ORCs) and directing the generated electricity to hydrogen production. Techno‐economic, life cycle, and environmental‐economic assessments show that this functional expansion can be achieved with minimal impact on natural gas affordability while favoring the ORC–PEMWE pathway.

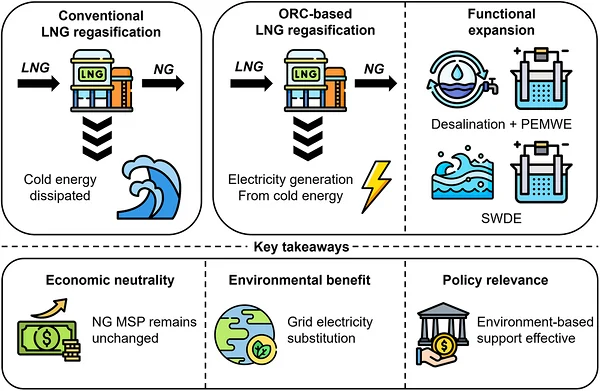

## 1 Introduction

Liquefied natural gas (LNG) plays a critical role in the global energy supply chain as a flexible and reliable energy carrier, particularly for regions with limited domestic energy resources. As the demand for LNG continues to increase, large‐scale LNG import terminals have become essential infrastructure, where regasification processes convert LNG from cryogenic storage conditions into gaseous natural gas (NG) suitable for pipeline transmission [1, 2].

During LNG regasification, a substantial amount of cryogenic energy is released as LNG is heated from cryogenic conditions (approximately −163°C) to near ambient conditions. The magnitude of this cold energy has been widely reported to be on the order of 830–860 kJ/kg‐LNG [3, 4], which corresponds to a significant cryogenic energy resource at the scale of modern LNG import terminals.

In conventional regasification systems, particularly those employing open‐rack vaporizers (ORVs), the cryogenic energy released during LNG vaporization is not recovered but is dissipated into the environment through large volumes of cooled seawater discharge [5, 6]. This practice has been reported to induce localized seawater temperature reductions, which may lead to environmental concerns in the vicinity of LNG terminals [7].

Beyond these local thermal effects, the dissipation of LNG cryogenic energy represents a significant system‐level opportunity loss from an energy and environmental perspective [3]. When unrecovered, the substantial temperature potential inherent to LNG regasification cannot be converted into useful electricity, thereby forfeiting opportunities for indirect emission reduction through displacement of externally supplied power.

Taken together, the systematic dissipation of LNG cryogenic energy during regasification constitutes not only an inefficiency in energy utilization but also an avoidable loss of low‐carbon energy potential. This observation motivates the investigation of cold energy recovery pathways that can be integrated into LNG terminals while preserving their primary function as natural gas supply infrastructure.

In response to this opportunity, numerous studies have investigated LNG cold energy utilization for power generation, energy storage, air separation, and hydrogen liquefaction processes. As summarized in Table 1, these studies primarily focus on improving thermodynamic performance and process or subsystem‐level economics by directly coupling cryogenic energy to specific applications. However, most existing analyses treat LNG cold energy as an externally available utility, while the implications for LNG terminal‐level economics and NG pricing remain largely unexplored.

**TABLE 1:** Summary of previous studies on LNG cold energy utilization and their analysis scope.

Refs.	Application	Cold energy coupling	Analysis scope	Economic evaluation	Environmental evaluation	Natural gas price
[3]	ORC + cooling (CCP system)	Direct	Subsystem‐ level	Included	Not included	Not included
[8]	ORC + CO_2_ capture	Direct	Subsystem‐ level	Included	^a^Partially included	Not included
[9]	CO_2_ liquefaction	Direct	Process‐ level	Included	Not included	Not included
[10]	Liquid air energy storage	Direct	Subsystem‐ level	Included	Not included	Not included
[11]	CES + ORC + direct cooling	Direct	Subsystem‐ level	Included	Not included	Not included
[12]	LAES + ASU multi‐generation	Direct	Subsystem‐ level	Included	Not included	Not included
[13]	ASU	Direct	Subsystem‐ level	Included	Not included	Not included
[14]	ASU	Direct	Process‐ level	Included	Not included	Not included
[15]	Hydrogen liquefaction	Direct	Process‐ level	Included	Not included	Not included
[16]	Hydrogen liquefaction	Direct	Process‐ level	Included	Partially included	Not included
^b^This study	ORC + hydrogen production	Indirect	Terminal‐ Level	Included	Included	Included

^a^ Indicates indirect environmental relevance (e.g., CO_2_ capture) without a systematic life cycle assessment.

^b^ Electricity‐only operation is included as a limiting case; however, the ORC is treated as an intermediate conversion step in the overall system framework.

An alternative system‐level approach is to first convert LNG cold energy into electricity. Among available technologies, organic Rankine cycles (ORCs) have been widely proposed for cold energy recovery due to their suitability for cryogenic heat sources and operational flexibility [17, 18, 19, 20]. By transforming cryogenic energy into electricity, ORC‐based configurations decouple cold energy utilization from application‐specific liquefaction or separation processes, enabling more versatile integration within existing LNG terminal infrastructure [21, 22].

Electricity recovered from LNG cold energy can be allocated to multiple downstream energy services, including grid export or internal power consumption within LNG terminals. In this study, electricity‐driven hydrogen production via water electrolysis is introduced not as an independent system of interest, but solely as a representative electricity allocation pathway to examine how recovered power may be utilized without altering the primary role of LNG terminals as NG supply infrastructure.

Among available electrolysis technologies, proton exchange membrane water electrolysis (PEMWE) and seawater direct electrolysis (SWDE) are considered due to their practical relevance to coastal LNG terminals. PEMWE offers high efficiency and operational flexibility but requires high‐purity water, whereas SWDE has the potential to directly utilize seawater and thereby reduce system complexity by eliminating the desalination step. These two pathways are therefore employed not to assess hydrogen production itself, but to represent distinct electricity utilization options that introduce different techno‐economic and environmental implications at the terminal level.

Accordingly, evaluating these electricity‐utilization pathways requires an integrated system‐level framework that connects process design with economic and environmental performance. Such approaches have recently been applied to hydrogen infrastructure, sustainable fuels, carbon management, and other low‐carbon conversion pathways [23, 24, 25, 26, 27, 28].

By conducting a terminal‐level techno‐economic analysis (TEA) and life cycle assessment (LCA), this work examines whether LNG cold energy recovery can be implemented without adversely affecting NG supply costs, while identifying system configurations that can enhance the overall energy efficiency and environmental performance of LNG regasification infrastructure. Rather than positioning hydrogen as a co‐primary output, hydrogen production is treated as a derived function enabled by recovered electricity, serving to illustrate potential secondary system benefits arising from cold energy utilization.

Through an environmental techno‐economic assessment (eTEA) of ORC‐integrated LNG terminals with alternative electricity allocation pathways, including PEMWE and SWDE, this study systematically investigates how different electricity utilization strategies influence terminal‐level performance under policy‐relevant considerations, such as electricity displacement potential, environmental performance improvement, and system‐level value creation. By comparing these pathways, the analysis provides insights into which conditions and configurations allow cold energy recovery to contribute most effectively to improving the functional efficiency and system value of LNG terminals within evolving low‐carbon energy systems.

## 2 Methods

### 2.1 Process Description

Figure 1 presents the overall process flowchart of the LNG regasification system and the downstream integrated units considered in this study. The system consists of a conventional ORV configuration and alternative configurations incorporating an ORC and optional hydrogen production processes. Across all configurations, the LNG throughput and NG delivery conditions are kept identical to ensure a consistent basis for comparison.

**FIGURE 1:**
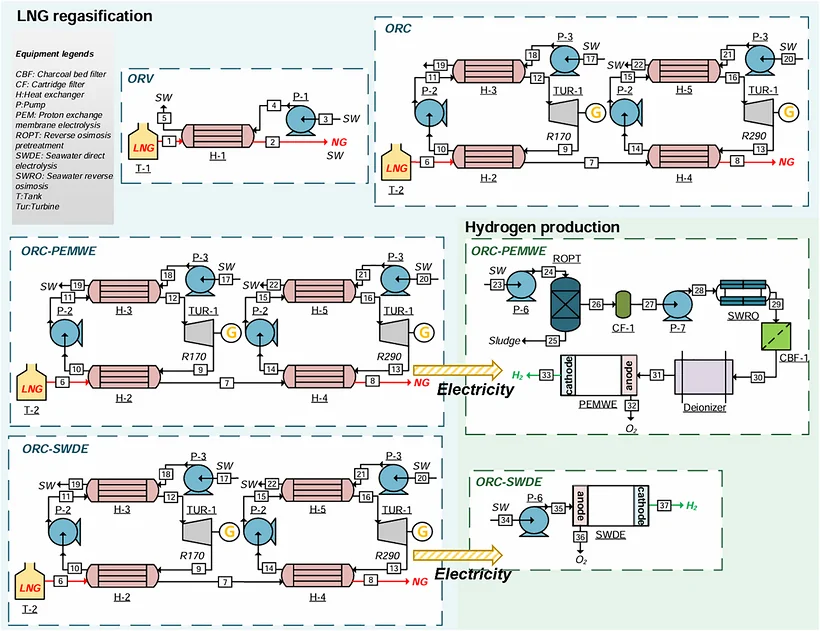
Overall process flow diagram of the LNG regasification system and integrated cold energy utilization pathways considered in this study.

In the conventional configuration (ORV), LNG is regasified using an open rack vaporizer, where seawater provides the thermal energy required for phase change and heating of LNG to the delivery conditions. All electricity required for auxiliary equipment in this configuration is supplied from external sources.

In the alternative configurations (ORC, ORC–PEMWE, and ORC–SWDE), the LNG regasification process is integrated with an ORC system to recover cold energy during regasification. In these configurations, thermal exchange between LNG, seawater, and the ORC working fluids enables electricity generation, which is either utilized internally by downstream units or exported, depending on the configuration.

For selected configurations, additional hydrogen production units are integrated downstream of the regasification process. The ORC–PEMWE configuration includes a seawater desalination unit followed by a PEMWE system for hydrogen production. In contrast, the ORC–SWDE configuration integrates a SWDE system, eliminating the need for a separate desalination step. These downstream units are supplied with electricity generated by the ORC system and are operated under the same LNG throughput and boundary conditions as the regasification process.

#### 2.1.1 Base Case

In the base configuration (ORV), LNG is withdrawn from the storage tank (T‐1) at −163°C and 1.1 bar and pressurized to 73 bar before entering the ORV (H‐1). The pressurized LNG is then regasified in the ORV using seawater as the heat source. Seawater enters the system at 20°C and 1 bar and is pressurized to 1.5 bar by a seawater pump prior to being supplied to the ORV (H‐1), where heat exchange with the LNG stream occurs. Through this heat transfer process, LNG is vaporized and heated to the delivery conditions.

At the outlet of the regasification process, NG is delivered at 10°C and 73 bar, meeting pipeline transmission requirements. The seawater exits the ORV at 15°C and 1.5 bar after releasing thermal energy to the LNG stream.

#### 2.1.2 Alternative Case

In the alternative configurations (ORC, ORC–PEMWE, and ORC–SWDE), the conventional LNG regasification process is integrated with two parallel ORC loops to enhance the recovery of LNG cold energy and generate electricity. As in ORV, LNG is supplied from the storage tank at −163°C and 1.1 bar and is pressurized to 73 bar prior to entering the regasification heat exchangers.

During preliminary process development, R170, R1270, R290, and R600a were considered as hydrocarbon working fluids spanning different phase change temperature levels. The candidate fluids were screened using their saturation temperatures at the fixed turbine outlet pressure of 1.1 bar, calculated from the Antoine equation parameters reported in the NIST Chemistry WebBook [29]. The turbine outlet pressure was specified before the operating condition sensitivity analyses.

R170 exhibited a saturation temperature approximately 41°C lower than that of R1270, the next lowest candidate, and was therefore assigned to the first ORC loop to represent the lowest phase change temperature level.

R1270 and R290 exhibited similar intermediate saturation temperatures, differing by approximately 5.6°C whereas R600a was classified as a separate higher temperature candidate. R1270 and R290 were therefore shortlisted as candidates for the second ORC loop. Both fluids have also been identified as suitable working fluids for LNG cold energy ORCs operating below ambient temperature in previous screening studies [30].

R290 was retained as the representative working fluid for the second ORC loop because it represents the higher saturation‐temperature member of the intermediate‐temperature group. This selection was not based on a direct comparative performance optimization between R290 and R1270. Accordingly, R170 and R290 represent the working fluids adopted for the selected ORC configuration rather than a globally optimal working fluid combination. The quantitative screening criteria and thermophysical properties of the candidate working fluids are summarized in Table SA.1.

In the first ORC loop, the working fluid R170 is fully condensed through heat exchange with LNG in heat exchanger H‐2. The condensed working fluid is then pressurized to 20 bar by pump P‐2 and subsequently vaporized in heat exchanger H‐3 using seawater as the heat source. The vaporized working fluid expands through turbine TUR‐1, producing electricity. After expansion, the working fluid is routed back to H‐2, where it exchanges heat with LNG and is recondensed, completing the first ORC cycle.

The second ORC loop operates in parallel following the same principle. In this loop, R290 is condensed in H‐4 via heat exchange with LNG, pressurized to 7 bar by pump P‐4, and vaporized in H‐5 using seawater. Electricity is generated through turbine TUR‐2, after which the working fluid returns to H‐4 to exchange heat with LNG, forming a closed‐loop cycle. The two parallel ORC loops were adopted as the base case process configuration to assign different working fluids to the lower and higher temperature portions of LNG regasification.

For ORC‐PEMWE and ORC‐SWDE, hydrogen production units are integrated downstream of the ORC system to utilize the electricity generated from the recovered cold energy. In ORC‐PEMWE, a seawater desalination unit followed by PEMWE is employed, where desalinated water is supplied to the electrolyzer for hydrogen production. In ORC‐SWDE, a SWDE system is considered, eliminating the need for a separate desalination step.

### 2.2 Process Modeling and Operating Conditions

Process simulations were performed using the Aspen Plus process simulator V15 under steady‐state conditions. The system, including conventional LNG ORV‐based regasification and the ORC‐based regasification, was modeled to evaluate mass and energy balances based on consistent operating assumptions. The hydrogen production system, including the desalination system, was not explicitly modeled in Aspen Plus but was evaluated based on literature‐reported performance indicators and electricity consumption data [31, 32, 33]. The specific energy consumption (SEC) values adopted for PEMWE and SWDE were 57 and 56 kWh/kg‐H_2_, respectively, based on Refs. [32, 33].

Key operating conditions and equipment performance assumptions are summarized in Table 2. The LNG inlet temperature and pressure were set to −163°C and 1.1 bar, respectively, while seawater was supplied at 20°C and near atmospheric pressure. At the outlet of the regasification process, NG was delivered at 10°C and 73 bar, consistent with high pressure transmission requirements. The outlet seawater temperature was fixed at 15°C, reflecting typical environmental discharge constraints.

**TABLE 2:** Simulation parameters (Aspen Plus V15).

Category	Parameter	Value
Inlet condition	LNG temperature (°C)	−163
	LNG pressure (bar)	1.1
	Seawater temperature (°C)	20
	Seawater pressure (bar)	1.1
Outlet condition	NG temperature (°C)	10
	NG pressure (bar)	73
	Seawater temperature (°C)	15
	Seawater pressure (bar)	1.5
Equipment	^a^Pump isentropic efficiency	0.75
	^a^Turbine isentropic efficiency	0.80
	Minimum temperature approach in heat exchangers (°C)	5

^a^ Although polytropic efficiency values are reported in [30], the same numerical values were conservatively applied as isentropic efficiencies, resulting in a conservative estimation.

For simulation, isentropic efficiencies of 0.75 for pumps and 0.80 for turbines were assumed [30]. A minimum temperature approach of 5°C was uniformly applied to all heat exchangers to ensure feasible heat transfer and conservative thermal design. These assumptions were consistently applied across all simulated cases to enable a fair comparison of system configurations.

The operating conditions of the two ORC loops were determined through sensitivity analyses in Aspen Plus. The pump discharge pressure and working fluid mass flow rate were selected as the decision variables.

For the R170 loop, the pump discharge pressure and working fluid mass flow rate were examined over 5–25 bar and 20–25 kg/s, respectively. No converged process solution was obtained at the tested pressures above 20 bar under the fixed model specifications. Therefore, 20 bar was retained as the highest converged pressure. The selected operating conditions were 20 bar and 24 kg/s. For the R290 loop, the pump discharge pressure and working fluid mass flow rate were examined over 1.2–10 bar and 30–35 kg/s, respectively. The selected operating conditions were 7 bar and 30.8 kg/s. The examined ranges and selected operating conditions for both ORC loops are summarized in Table SA.2.

For each loop, the operating point providing the highest loop net power output among the converged sensitivity cases was selected. The loop net power output was calculated by subtracting the power consumption of the corresponding working fluid and seawater pumps from the turbine power output. The turbine outlet pressure and minimum temperature approach were fixed at 1.1 bar and 5°C, respectively. Unless otherwise specified, pressure drops in heat exchangers and auxiliary units were neglected, and all simulations were conducted under steady‐state conditions without considering transient effects.

### 2.3 Techno‐Economic Analysis

#### 2.3.1 Economic Assumptions

The TEA was implemented as a process‐based assessment that links simulated mass and energy balances to equipment costs, operating expenditures, product revenues, and financial assumptions. Similar process‐based economic and environmental assessments have been used for low‐carbon conversion and resource upgrading systems [34, 35, 36, 37, 38], as well as waste‐gas utilization, biomass and microalgal conversion, municipal solid‐waste upcycling, and integrated co‐production systems [39, 40, 41, 42, 43, 44].

All cost estimates were calculated in 2024$. When cost data from other base years were used, costs were updated to 2024$ using the Chemical Engineering Plant Cost Index (CEPCI), following:

$$C_{2024}=C_{base\;year}\;\times \frac{CEPCI_{2024}}{CEPCI_{base\;year}}$$

The economic parameters listed in Table 3 were selected to represent typical financial assumptions for large‐scale energy infrastructure projects. These parameters were applied consistently across all systems. Operation rate reflects the fraction of annual time during which the system operates at the specified design conditions, accounting for scheduled maintenance and non‐continuous terminal operation. Startup conditions were explicitly considered by assuming reduced revenue and partial variable cost recovery during the startup period. The unit prices, annual operating assumptions, and CEPCI values used in the TEA are summarized in Table SB.1.

**TABLE 3:** Major economic assumptions used in TEA [45].

Plant financing by equity (%)	40
Plant life (years)	30
Income tax rate (%)	21
Internal rate of return (%)	10
General plant depreciation (years)*	7
Working capital (% of fixed capital investment)	5
Operation rate (%)	90
Startup time (years)	0.5
Revenue and costs during startup	
Revenue (% of normal)	50
Variable costs (% of normal)	70
Fixed costs (% of normal)	100
Construction period (years)	3

#### 2.3.2 Capital Expenditure (CAPEX) Estimation

The total installed CAPEX for each configuration was calculated by aggregating section‐level capital costs for LNG regasification, ORC, seawater desalination (where applicable), and hydrogen production units. Cost estimation methods for each section are summarized below.

1. LNG regasification and ORC sections: For the ORV‐based regasification (ORV) and ORC integrated configurations (ORC, ORC–PEMWE, ORC–SWDE), equipment purchase and installed costs for the LNG regasification and ORC sections were obtained using Aspen Economic Analyzer. The process flow diagrams and operating conditions from the Aspen simulations were used to size major equipment items (e.g., heat exchangers, pumps, turbines). The resulting equipment‐level installed costs were summed up to obtain the section‐level CAPEX for LNG regasification and ORC integration.
2. Seawater desalination unit: For ORC–PEMWE, the seawater desalination unit was treated as a packaged subsystem. Its CAPEX was estimated using a literature‐reported capital cost correlation expressed as a function of plant capacity ($/MLD) [31]. The required desalination capacity was determined from the electrolyzer water demand, and the total installed CAPEX was calculated assuming linear scaling with plant capacity.
3. Water electrolysis: For ORC–PEMWE, the PEMWE system CAPEX was estimated using literature values reported on a $/kW basis [32]. The required electrolyzer capacity was calculated from the available ORC electricity and the specified specific energy consumption. The total installed CAPEX was then obtained by assuming linear scaling with electrolyzer capacity, consistent with common practice for modular electrolyzer systems. For ORC‐SWDE, commercial‐scale cost data were not available. The SWDE CAPEX was therefore represented using a scenario multiplier relative to the PEMWE system at an equivalent power capacity. A multiplier of 2.0 was applied as a scenario assumption. This multiplier was not derived from a specific literature source and should not be interpreted as a commercially established SWDE cost estimate. Its influence on the economic results was evaluated through the sensitivity analysis presented in Section 3.1.3. Detailed capital cost worksheets for the ORV, ORC, ORC‐PEMWE, and ORC‐SWDE configurations are provided in Tables SB.2–SB.5.

#### 2.3.3 Operating Expenditures (OPEX)

Fixed operating expenditures were estimated as a function of capital investment, with labor costs assumed as 1.5% of the fixed capital investment (FCI), maintenance costs as 3.0% of the inside battery limits capital cost, and property‐related costs as 0.7% of FCI.

Variable operating expenditures were calculated based on process specific material and utility consumption and are summarized in Table SB.1. These include feedstock costs, electricity consumption, and subsystem specific operating costs associated with desalination and electrolysis units.

Component durability was incorporated into the replacement‐related operating costs of the electrolysis systems. The replacement interval of PEMWE was set to 40 000 operating hours based on ref. [32]. For SWDE, a replacement interval of 500 operating hours was conservatively assumed based on the experimentally demonstrated durability reported in ref. [33]. This value should therefore be interpreted as a modeling assumption based on the demonstrated operating period rather than as a commercially established SWDE lifetime. Accordingly, the replacement‐related operating cost of SWDE was estimated by applying the 80‐fold replacement frequency ratio relative to PEMWE.

#### 2.3.4 Minimum Selling Price

The economic performance of each system was evaluated using the minimum selling price (MSP) of NG. The MSP is defined as the NG price at which the net present value (NPV) of the project becomes zero over the plant lifetime.

Annual cash flows were calculated by accounting for capital recovery, fixed and variable operating expenditures, taxes, and revenues from byproducts. Revenues from electricity export and hydrogen production were treated as byproduct credits and deducted from the total annual cost. The NPV was calculated according to:

$$PV=\sum_{t=1}^{N}\frac{CF_{t}}{{\left(1+r\right)}^{t}}$$

where CF_t_ represents the net cash flow in year t, r is the internal rate of return, and N is the plant lifetime. The MSP of NG was iteratively determined to satisfy NPV = 0 under the economic assumptions summarized in Table 3. The complete discounted cash flow calculations for the ORV, ORC, ORC‐PEMWE, and ORC‐SWDE configurations are provided in Tables SB.6–SB.9.

### 2.4 Life Cycle Assessment

LCA was employed to quantify the environmental impacts of the proposed LNG regasification systems and to identify major environmental contributors. The assessment was conducted in accordance with the guidelines of the International Organization for Standardization [46, 47] and consisted of four main steps: (1) goal and scope definition, (2) inventory analysis, (3) impact assessment, and (4) interpretation.

1. Goal and scope definition: The goal of this study was to evaluate the environmental impacts of a conventional LNG regasification process and ORC‐integrated regasification systems with hydrogen co‐production. The system boundary was defined on a gate‐to‐gate basis, encompassing the LNG regasification process and downstream integrated units, as illustrated in Figure 2. Processes related to LNG production, liquefaction, and transportation were excluded. The functional unit was defined as 1 kg‐NG at the outlet of the regasification process, allowing a consistent comparison among all system configurations. Recent sustainability and LCA studies further emphasize the importance of comprehensive life cycle coverage, explicit system boundary definition, and transparent selection between attributional and consequential modeling [48, 49, 50, 51, 52].
2. Inventory analysis: Based on the defined functional unit, all relevant material and energy flows crossing the system boundary were quantified. Life cycle inventory data were primarily obtained from mass and energy balances calculated using Aspen Plus simulations. Background inventory data for auxiliary inputs were sourced from the ecoinvent database (version 3.12). The foreground input/output inventories and the corresponding background datasets are provided in Tables SC.1 and SC.2, respectively. In addition to the NG‐based functional unit, the same integrated system was also evaluated using hydrogen as the functional unit (1 kg‐H_2_), where NG was treated as an avoided product. The inventory calculation followed the same system boundary and methodological framework.
3. Impact assessment: Environmental impacts were calculated using the Environmental Footprint (EF) version 3.1 impact assessment method. Multifunctionality arising from the co‐production of NG, electricity, and hydrogen was addressed by applying system expansion by substitution, in line with ISO 14040 and ISO 14044 [46, 47]. Exported electricity and co‐produced hydrogen were assumed to replace conventionally produced electricity and hydrogen on an equivalent‐output basis, corresponding to a 1:1 substitution using the respective ecoinvent datasets listed in Table SC.2. The corresponding avoided environmental burdens were credited to the system. Among the evaluated impact categories, particular attention was given to global warming potential (GWP).
4. Interpretation: The LCA results were analyzed to identify key contributors to environmental impacts and to assess the influence of system integration on overall environmental performance. The interpretation emphasized methodological consistency, transparency, and robustness.

**FIGURE 2:**
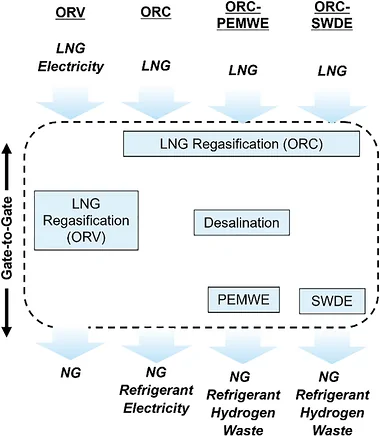
LCA boundary of ORV, ORC, ORC–PEMWE, ORC–SWDE (gate‐to‐gate).

### 2.5 Environmental Techno‐Economic Assessment

An eTEA was performed to integrate the environmental performance obtained from LCA with the economic evaluation of the LNG regasification systems. This joint interpretation is consistent with recent eTEA applications that evaluate cost and environmental indicators within a common process‐system framework [25, 53]. The assessment was conducted in accordance with ISO 14076 [54], which provides guidance on the valuation of greenhouse gas emissions for decision‐making purposes.

The GWP results from the LCA were converted into economic terms by applying carbon credits based on region‐specific carbon price assumptions. The resulting environmental benefits were treated as additional revenues and incorporated into the calculation of the environmental cost‐added minimum selling price (eMSP) of NG.

A sensitivity analysis was carried out by varying key policy related parameters, including carbon credit levels and CAPEX support rates, to assess their influence on the economic feasibility of the proposed systems.

#### 2.5.1 Carbon Credit

Carbon credits were incorporated to monetize greenhouse gas emission reductions achieved by the proposed systems. Country specific carbon credit prices were applied, as summarized in Table 4. The carbon credit benefit was calculated as the product of the GWP reduction relative to the reference case and the corresponding carbon credit price.

**TABLE 4:** Carbon credit for each region.

Region	Carbon price ($/ton)	Ref.
Korea	6.9	[55]
China	12.8	
USA (California)	37.9	
Europe	65.5	

The resulting credit was treated as an operating stage incentive and implemented as an additional revenue term in the economic assessment. Accordingly, carbon credits were reflected in the OPEX balance when calculating the eMSP of NG.

#### 2.5.2 CAPEX Grant

CAPEX support was introduced as a scenario‐based investment incentive to evaluate the sensitivity of the system economics to capital‐level policy support. In this study, the CAPEX grant was applied to the total capital cost of the integrated system, including LNG regasification, ORC, and downstream hydrogen production units. The grant amount was calculated as the product of the total CAPEX and an assumed subsidy rate.

Although investment support schemes for clean hydrogen are often associated with hydrogen production facilities, this study adopted a system‐level support assumption, reflecting the integrated nature of the proposed configuration in which LNG regasification, power generation, and hydrogen production operate as a single coupled system. Accordingly, the CAPEX reduction was applied uniformly to the entire system rather than being restricted to individual subunits.

The reduced capital cost was annualized using the same economic assumptions applied in the base TEA and incorporated into the calculation of the eMSP of NG.

## 3 Results and Discussion

### 3.1 Techno‐Economic Analysis

#### 3.1.1 Equipment and Operating Costs

Figure 3 presents the incremental annualized capital and operating cost changes of the ORC‐based configurations relative to the ORV base case. To enable a meaningful comparison of cost variations associated with system integration, the LNG feedstock cost, which is common to all configurations, is excluded from Figure 3. The total annual cost increases from 1531 million $/yr for ORV to 1540, 1635, and 1871 million $/yr for ORC, ORC–PEMWE, and ORC–SWDE, respectively, reflecting the additional process units integrated beyond conventional LNG regasification.

**FIGURE 3:**
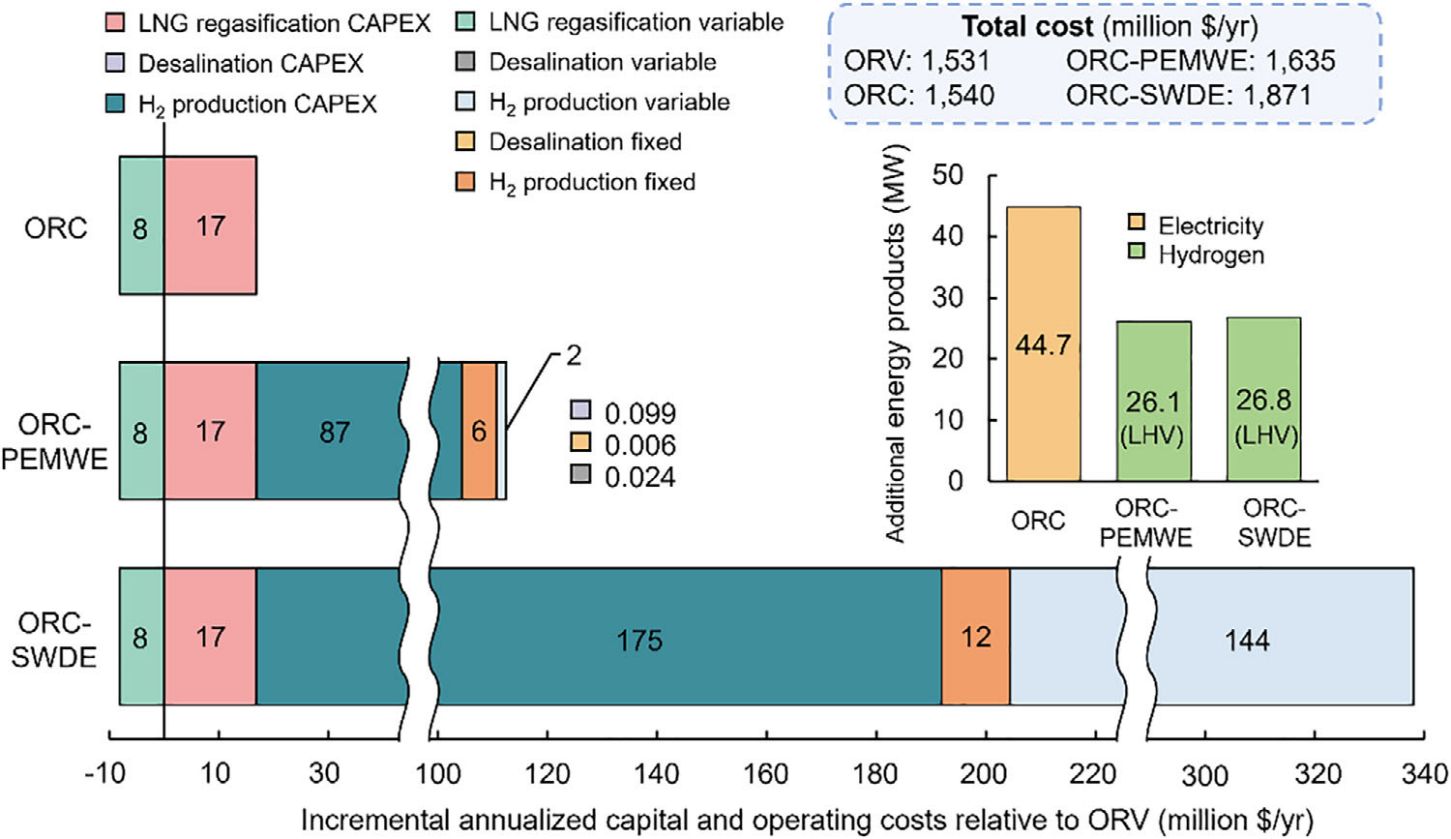
Incremental costs and additional energy outputs of ORC integrated LNG regasification systems relative to the ORV.

From the LNG regasification perspective, the introduction of the ORC system leads to a net increase in capital expenditure, primarily associated with the installation of turbines, pumps, and heat exchangers required for cold energy recovery. This capital increase is consistently observed across all ORC‐based configurations. In contrast, the fixed operating cost associated with electricity consumption in the regasification section decreases relative to ORV, as part of the externally purchased electricity is substituted by internally generated power from the ORC system. As shown in Figure 3, this electricity‐related cost appears as a negative contribution when expressed on an incremental basis. At the terminal scale, the R170 and R290 turbines produced a total power output of 60.67 MW. After subtracting the total pump power consumption of 15.97 MW, the terminal net power output was calculated to be 44.70 MW. The corresponding terminal‐level thermodynamic performance indicators, including heat exchanger duties, net thermal efficiency, and specific net power output, are summarized in Table SD.1.

For the ORC–PEMWE configuration, the hydrogen production section introduces additional capital and operating costs; however, its overall contribution remains limited relative to the scale of LNG regasification. At an LNG throughput of 2 160 000 kg/h, the corresponding hydrogen production rate is approximately 783 kg‐H_2_/h, supported by 7049 kg/h of desalinated water. This auxiliary hydrogen output represents a functional expansion of the LNG terminal rather than a structural change in system scale and therefore has a limited influence on the total annual cost profile.

In contrast, the ORC–SWDE configuration exhibits a substantially larger incremental cost contribution from the hydrogen production section. The total annual cost of ORC–SWDE was calculated to be 1871 million $/yr, compared with 1635 million $/yr for ORC–PEMWE. This increase is primarily attributable to the replacement‐related operating cost associated with the conservative 500 h replacement interval assumed for SWDE, together with the higher initial CAPEX represented by the 2.0 multiplier relative to PEMWE. Accordingly, the elevated cost of ORC–SWDE reflects the combined effects of the durability assumption and the prospective capital cost assumption, rather than the initial CAPEX alone.

#### 3.1.2 Minimum Selling Price of Natural Gas

Figure 4 presents the MSP of NG calculated for the four configurations. The MSP values were 324.5, 323.2, 324.6, and 334.6$/ton‐NG for ORV, ORC, ORC–PEMWE, and ORC–SWDE, respectively. When ordered from the lowest to the highest MSP, the configurations were ranked as ORC, ORV, ORC–PEMWE, and ORC–SWDE. Relative to ORV, the MSP decreased by approximately 0.4% for ORC, remained nearly unchanged for ORC–PEMWE, and increased by approximately 3.1% for ORC–SWDE.

**FIGURE 4:**
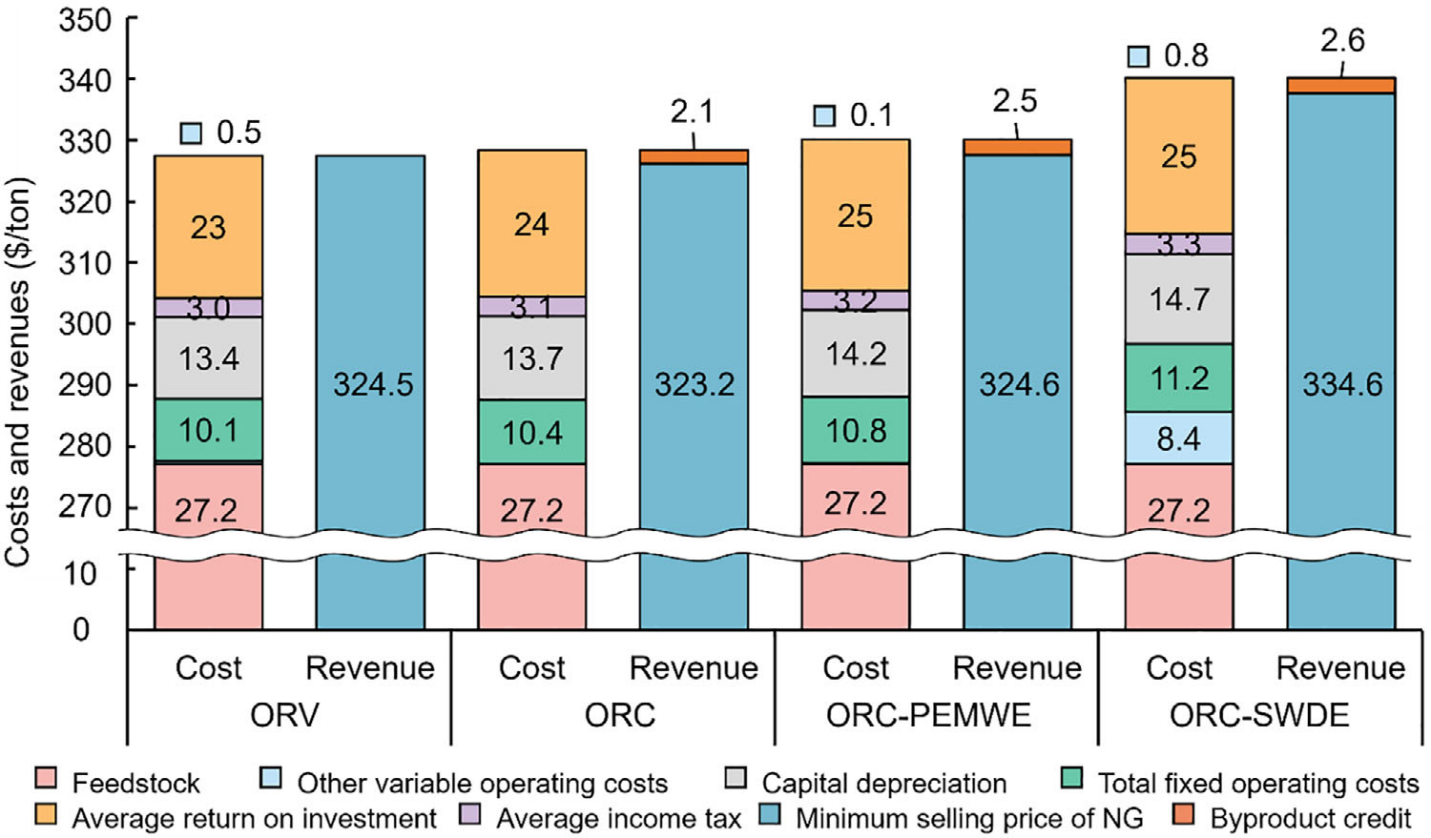
Costs and revenues for each case.

The MSPs of ORC and ORC–PEMWE remain close to those of conventional ORV because the LNG feedstock cost continues to dominate the overall cost structure. The electricity generated by the ORC offsets the additional capital and operating costs of cold energy recovery, while hydrogen revenue in ORC–PEMWE largely compensates for the cost of desalination and electrolysis. In contrast, the replacement‐related operating cost assumed for SWDE is sufficiently large to produce a more distinct increase in MSP despite the additional hydrogen revenue.

The ORC configuration exhibits a slightly lower MSP than the conventional ORV configuration. This difference is associated with the additional electricity generated through LNG cold energy recovery, which provides a modest revenue stream that partially offsets the additional capital investment required for ORC installation. In contrast, the ORV configuration relies entirely on externally purchased electricity, without generating any byproduct revenue from power production.

The ORC–PEMWE and ORC–SWDE configurations further introduce hydrogen as an additional byproduct. In ORC–PEMWE, hydrogen revenue largely offsets the additional capital and operating costs, resulting in an MSP of 324.6$/ton‐NG, which is nearly identical to that of ORV. In contrast, the hydrogen revenue generated in ORC–SWDE is insufficient to compensate for the substantially higher replacement‐related operating cost assumed for the SWDE system. Consequently, the MSP of ORC–SWDE increases to 334.6$/ton‐NG.

Overall, the results demonstrate that ORC integration and the ORC–PEMWE pathway can be implemented without materially increasing the NG selling price. The ORC configuration slightly reduces the MSP, while ORC–PEMWE maintains economic parity with conventional regasification despite the addition of hydrogen production. By contrast, ORC–SWDE exhibits a moderate economic penalty under the current replacement interval and capital cost assumptions. These results indicate that the economic feasibility of functional expansion depends strongly on the durability and technological maturity of the downstream system.

#### 3.1.3 Economic Sensitivity Analysis

Figure 5 presents the results of the economic sensitivity analysis, in which key parameters were varied by ±20% to evaluate their impact on the MSP of NG. The red horizontal line represents the baseline MSP of ORV, serving as a reference for comparison across all cases.

**FIGURE 5:**
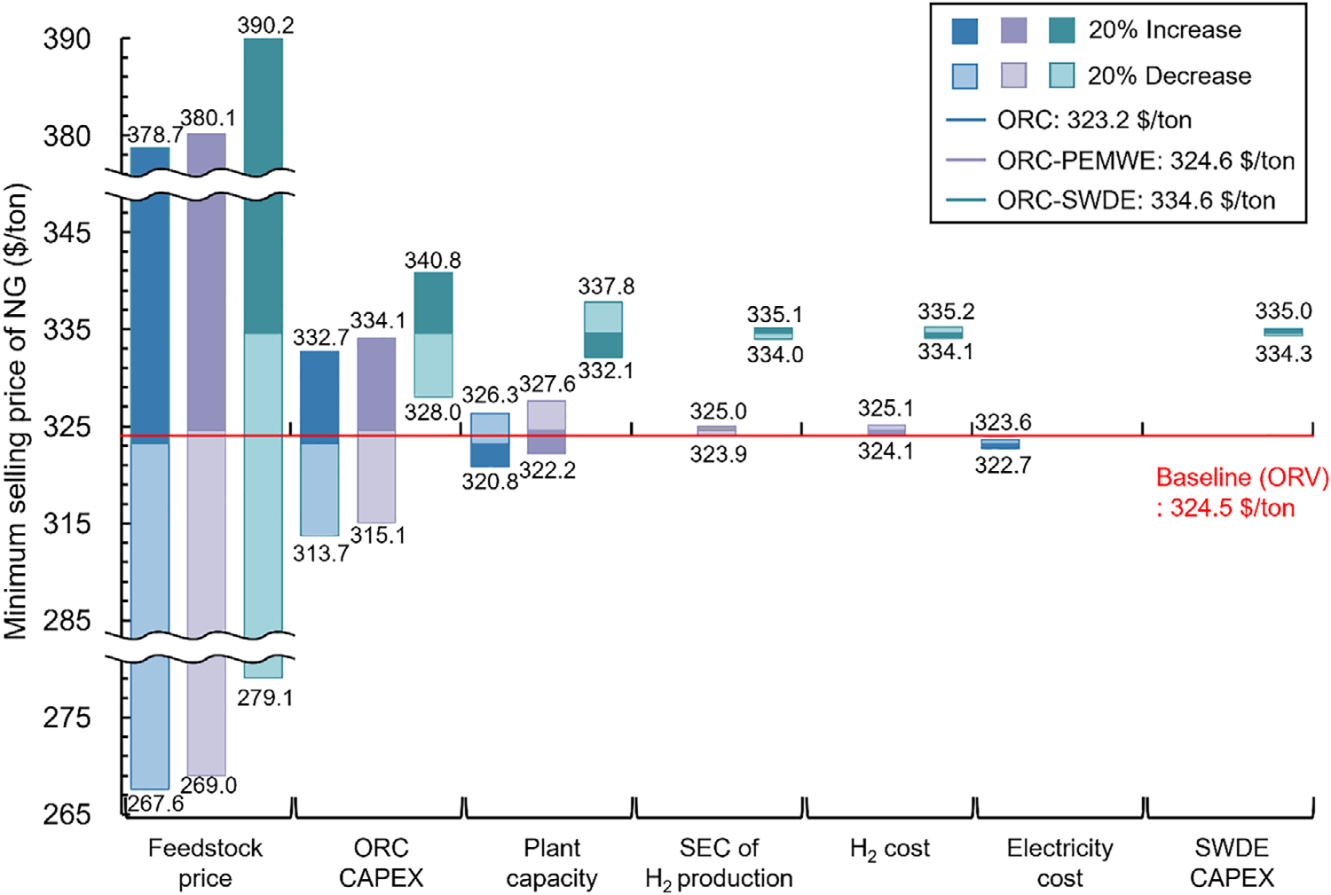
Sensitivity analysis for key economic and operating parameters.

Among the parameters considered, the LNG feedstock price exhibits the largest influence on MSP for all cases. This result reflects the dominant role of feedstock cost in the overall NG price structure, as discussed in the previous sections. Because the feedstock price is determined and uniformly applied across all configurations, the sensitivity results indicate that variations in MSP are largely driven by market conditions rather than by differences in system configuration or process integration.

Following the feedstock price, the CAPEX associated with the ORC system shows the second largest impact on MSP in ORC‐integrated cases. A ±20% variation in ORC CAPEX leads to a noticeable shift in MSP, highlighting the importance of capital investment in cold energy recovery systems. Under reduced ORC CAPEX conditions, the ORC and ORC–PEMWE configurations achieve MSP values comparable to, or slightly lower than, the ORV baseline. This result suggests that ORC‐based integration can maintain economic parity with conventional regasification when capital costs are moderated, rather than requiring fundamental changes in feedstock or operating conditions.

Plant capacity also has a clear effect on MSP, with larger capacities resulting in lower MSP values. This trend is primarily driven by economies of scale, whereby capital costs are distributed over increased throughput.

The SEC of hydrogen production has a relatively limited influence on NG MSP. A 20% variation in SEC changes the MSP from 323.9 to 325.0$/ton‐NG for ORC–PEMWE and from 334.0 to 335.1$/ton‐NG for ORC–SWDE. A reduction in SEC allows the MSP of ORC–PEMWE to fall slightly below the ORV baseline, whereas ORC–SWDE remains above the baseline throughout the examined range. These results indicate that improvements in electrolysis efficiency alone are insufficient to overcome the replacement‐related operating cost assumed for SWDE.

To evaluate the uncertainty associated with this scenario assumption, the economic sensitivity analysis was expanded to include the SWDE CAPEX. A ±20% variation around the base case multiplier corresponds to SWDE CAPEX values ranging from 1.6 to 2.4 times the PEMWE CAPEX. Over this range, the NG MSP of ORC–SWDE varied from 334.3 to 335.0$/ton‐NG. The relatively limited response indicates that, within the evaluated range, the higher MSP of ORC–SWDE is governed primarily by the replacement‐related operating cost associated with the conservative 500 h replacement interval, rather than by the initial CAPEX assumption alone.

Overall, external market parameters directly associated with the primary function of the system, most notably the LNG feedstock price—remain the dominant drivers of the NG MSP. Parameters associated with auxiliary units, including ORC integration and hydrogen production, introduce incremental but systematic shifts in MSP. While these effects are bounded in magnitude due to the inherent scale difference between the LNG regasification process and the downstream energy conversion units, they represent additional value streams that are realized without altering the primary LNG throughput or operating conditions.

### 3.2 Life Cycle Assessment

#### 3.2.1 LNG Regasification

Figure 6a presents a comparative heatmap of normalized life cycle environmental impacts for the integrated LNG regasification system, which includes ORC integration and downstream hydrogen production where applicable. The results are normalized with respect to ORV, which is set as the reference (100%), allowing for a relative comparison of environmental performance among the different system configurations.

**FIGURE 6:**
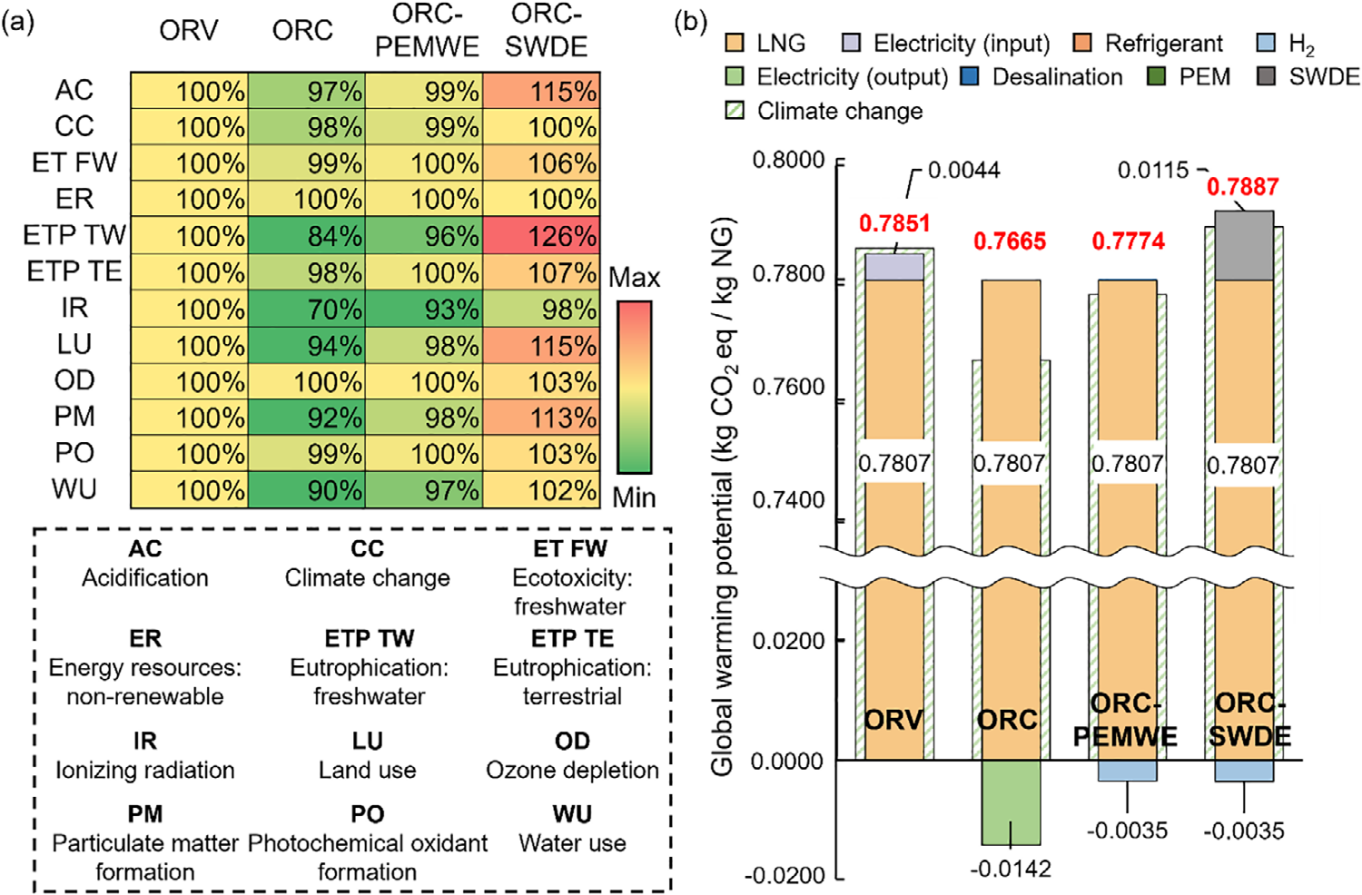
(a) Relative life cycle impact comparison across environmental categories; (b) GWP breakdown of LNG regasification systems.

Across most environmental impact categories, including climate change, acidification, eutrophication, and particulate matter formation, the integrated cases exhibit variations within a limited range compared to the reference case. Most impact indicators remain close to the baseline level, typically within ±10%, indicating that the incorporation of additional process units does not substantially alter the overall environmental profile of the system. The complete numerical results for all evaluated life cycle impact categories are provided in Table SC.3.

Notably, the integration of ORC leads to a consistent improvement in environmental performance across several key impact categories, particularly climate change. By recovering LNG cold energy, the ORC configuration achieves lower life cycle impacts than the ORV baseline, demonstrating that energy recovery at LNG terminals can deliver tangible environmental benefits rather than merely avoiding additional burdens.

The ORC–PEMWE configuration maintains this favorable trend while enabling hydrogen production as an additional energy carrier. Despite the introduction of downstream electrolysis and desalination units, the overall environmental impacts remain lower than or comparable to the baseline across most categories, including GWP. This indicates that hydrogen production, when powered by internally recovered energy, can be integrated into LNG terminals without offsetting the environmental gains achieved through ORC‐based energy recovery. In this sense, ORC–PEMWE represents a balanced configuration that combines functional expansion with net environmental improvement.

In contrast, the ORC–SWDE configuration exhibits higher impacts in several categories, including climate change, freshwater ecotoxicity, and water use. This increase is primarily attributed to the replacement‐related material burden associated with the conservative 500 h replacement interval and the corresponding material inventory assumed for SWDE. As a result, the additional environmental burdens associated with SWDE outweigh the benefits gained from cold energy recovery under the current assumptions.

As shown in Figure 6b, ORC exhibits the lowest GWP, followed by ORC–PEMWE and ORV, while ORC–SWDE shows the highest value among the evaluated configurations. The lower GWP observed in ORC can be attributed to the effective recovery of cold energy through ORC integration, which reduces the need for externally supplied electricity without introducing additional downstream processes. ORC–PEMWE shows a slightly higher GWP than ORC but remains comparable to the baseline, indicating that the inclusion of hydrogen production does not significantly deteriorate climate performance when appropriately integrated with energy recovery.

In contrast, ORC–SWDE exhibits the highest GWP, reflecting the short lifetime associated with the seawater direct electrolysis system. Although the increase in GWP remains moderate in absolute terms, ORC–SWDE represents a less favorable configuration from a climate change perspective under the current assumptions.

Overall, the bar chart analysis confirms that ORC achieves the most favorable climate performance, while ORC‐PEMWE represents a balanced configuration that enables functional expansion with only a marginal increase in GWP, and ORC–SWDE highlights the environmental trade‐offs associated with less mature downstream technologies.

#### 3.2.2 Sensitivity to Substitute Electricity Datasets

The environmental benefit assigned to exported electricity through system expansion depends on the data set used to represent the substituted electricity. To evaluate this uncertainty, the GWP of the ORC configuration was recalculated using alternative regional electricity market datasets and electricity production technology datasets. The functional unit, system boundary, impact assessment method, and 1:1 substitution ratio were maintained in all cases, while only the substituted electricity dataset was changed.

As shown in Figure 7a, the GWP of the ORC configuration ranged from 0.7487 to 0.7801 kg CO_2_‐eq/kg NG when regional electricity market datasets were applied. This variation reflects differences in the electricity supply mix represented for each region. Substitution of an electricity mix with a greater upstream greenhouse gas burden resulted in a larger environmental credit and a lower net GWP for the ORC configuration.

**FIGURE 7:**
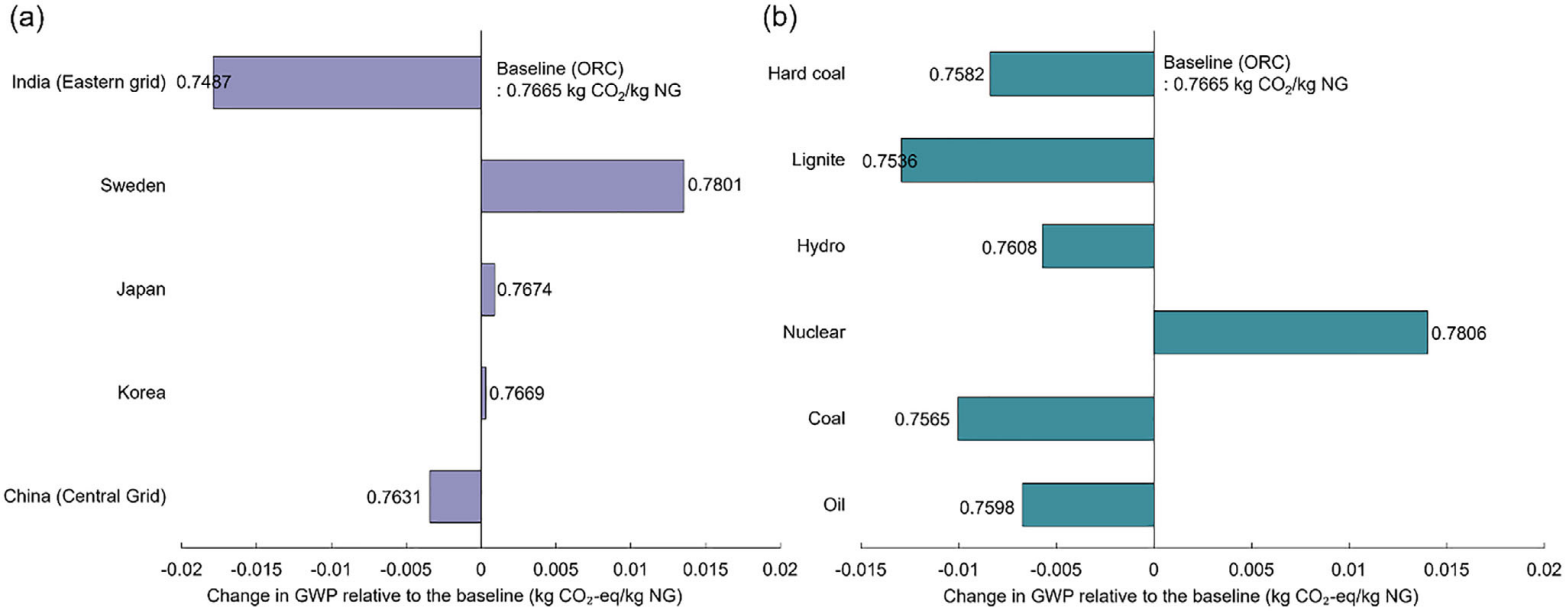
Sensitivity of the GWP of the ORC configuration to the substituted electricity dataset: (a) regional electricity market datasets and (b) electricity production technology datasets.

Electricity production technology also influenced the calculated environmental benefit. As shown in Figure 7b, the GWP of ORC ranged from 0.7536 to 0.7806 kg CO_2_‐eq/kg NG when alternative electricity production datasets were applied. These results indicate that the magnitude of the avoided burden depends not only on the geographic scope of the electricity market but also on the production technology assumed to be displaced.

Although the absolute GWP varied depending on the selected electricity dataset, all evaluated ORC scenarios remained below the ORV result of 0.7851 kg CO_2_‐eq/kg NG. Therefore, the magnitude of the environmental benefit is sensitive to the substituted electricity dataset, but the conclusion that ORC‐based LNG cold energy recovery reduces the GWP of the regasification system remains unchanged. The electricity datasets used in this analysis are provided in Table SC.4. An additional sensitivity analysis of the ORC‐PEMWE configuration to the geographic scope of the substituted hydrogen dataset is presented in Figure SC.1, with the corresponding hydrogen datasets also listed in Table SC.4.

#### 3.2.3 Hydrogen Production

To evaluate the environmental performance of hydrogen production within the integrated system, an LCA was conducted by defining NG as an avoided product. This approach reflects the fact that hydrogen is produced as an additional function enabled by system integration, while NG remains the primary product of the regasification process.

Based on this framework, the GWP of hydrogen produced in ORC–PEMWE is calculated to be 0.420 kg CO_2_‐eq/kg‐H_2_. This value is comparable to the life cycle emission ranges reported for green hydrogen produced via renewable‐powered electrolysis (typically ∼0.4–2 kg CO_2_‐eq/kg‐H_2_) and is lower than those of blue hydrogen produced from NG with carbon capture (approximately 2–5 kg CO_2_‐eq/kg‐H_2_), as reported in recent LCA studies [56, 57, 58]. These results demonstrate that low‐carbon hydrogen can be achieved through the proposed integrated LNG regasification system.

The low GWP of hydrogen in ORC–PEMWE can be attributed to the effective utilization of recovered cold energy and internally generated electricity, which significantly reduces reliance on carbon‐intensive external power sources. As a result, hydrogen production is achieved with minimal additional life cycle greenhouse gas emissions beyond those associated with the baseline NG system.

Importantly, the obtained GWP level falls well within the range required to meet low‐carbon hydrogen thresholds adopted in various policies and regulatory frameworks. The calculated value satisfies the most stringent tier of the U.S. Inflation Reduction Act (Section 45 V) and complies with the EU RFNBO requirements under RED II/III. This suggests that hydrogen produced under the conditions represented by ORC–PEMWE would be eligible for incentives such as preferential certification, tax exemptions, or capital support mechanisms aimed at promoting low‐carbon hydrogen deployment.

Overall, these results highlight the environmental viability of hydrogen production integrated with LNG regasification when appropriate system configurations are applied. The findings confirm that ORC–PEMWE enables the production of environmentally competitive hydrogen while maintaining consistency with both life cycle sustainability objectives and emerging policy criteria.

### 3.3 Environmental Techno‐Economic Assessment

#### 3.3.1 Environmental Cost‐Added Minimum Selling Price of Natural Gas

Figure 8 presents the eMSP of NG by incorporating carbon credit revenues derived from the reduction in GWP of the main product, NG. The analysis focuses on ORC and ORC–PEMWE, where system integration leads to lower GWP compared to the conventional regasification configuration.

**FIGURE 8:**
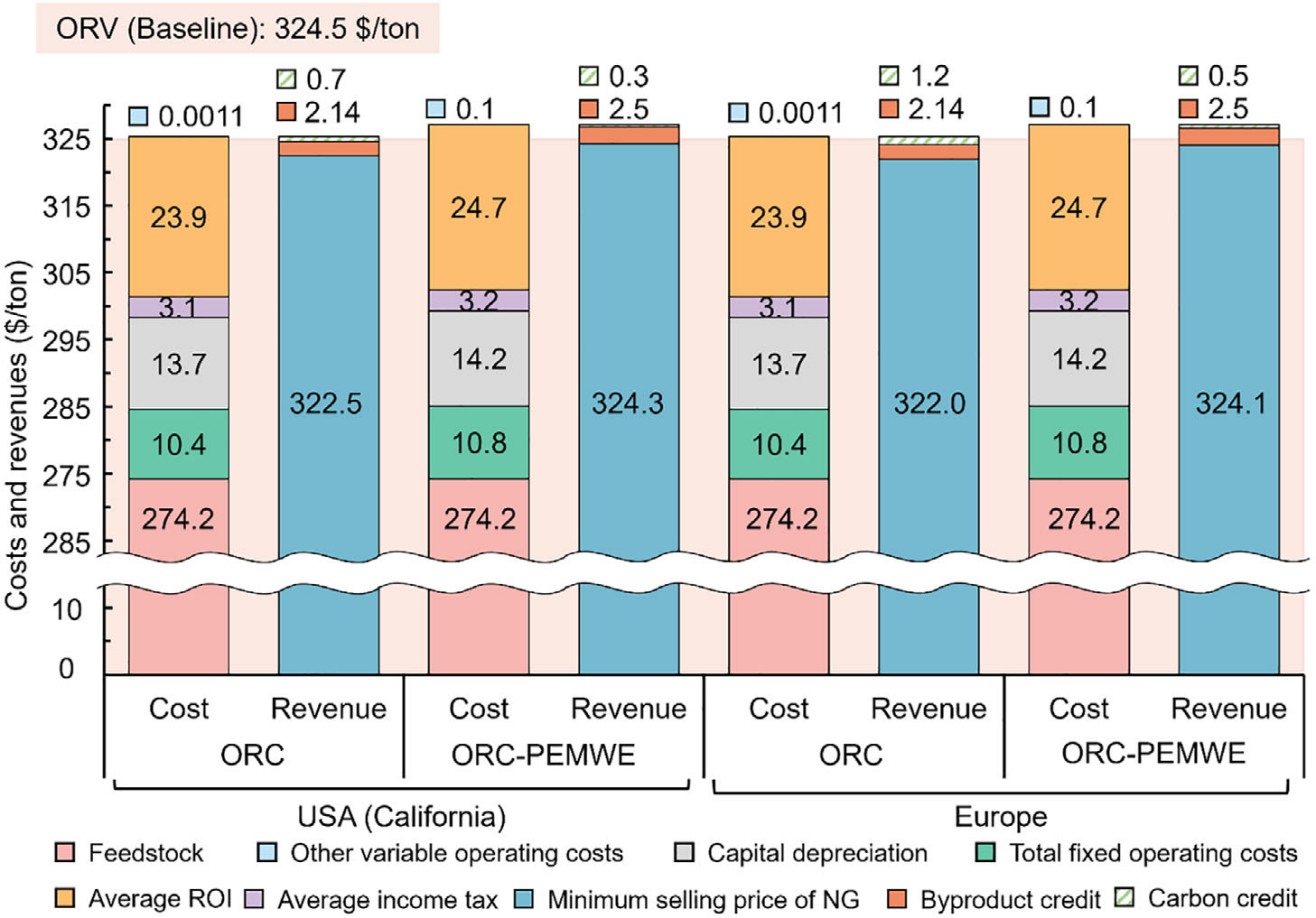
Environmental cost‐added costs and revenues for each case.

The baseline price corresponds to the MSP of NG obtained from the ORV, which does not benefit from carbon credit revenues. In contrast, the reduced GWP achieved in ORC and ORC–PEMWE enables the application of carbon credit schemes under regional policy frameworks in the United States (California) and Europe, resulting in additional economic benefits.

When carbon credits are accounted for, the eMSP of NG in the ORC‐integrated cases decreases below the baseline level. Specifically, the application of carbon credits yields additional revenues of 0.7 and 0.3$/ton‐NG in the United States for ORC and ORC–PEMWE, respectively, and 1.2 and 0.5$/ton‐NG in Europe. Although these values are modest, they indicate that improvements in environmental performance can be partially reflected in NG pricing under existing policy mechanisms.

This indicates that carbon credit revenues alone are insufficient to induce substantial changes in NG MSP and that the economic relevance of environmental improvements remains closely tied to the scope and strength of policy frameworks.

#### 3.3.2 Environmental Sensitivity Analysis

Figure 9a illustrates the environmental sensitivity analysis of ORC–PEMWE under policy support schemes for clean hydrogen, considering the combined effects of CAPEX grants and carbon credit levels on the MSP of NG. The heatmap represents the variation in NG eMSP as a function of CAPEX subsidy rate and carbon credit price, providing insight into the conditions under which environmental benefits can be translated into economic advantages.

**FIGURE 9:**
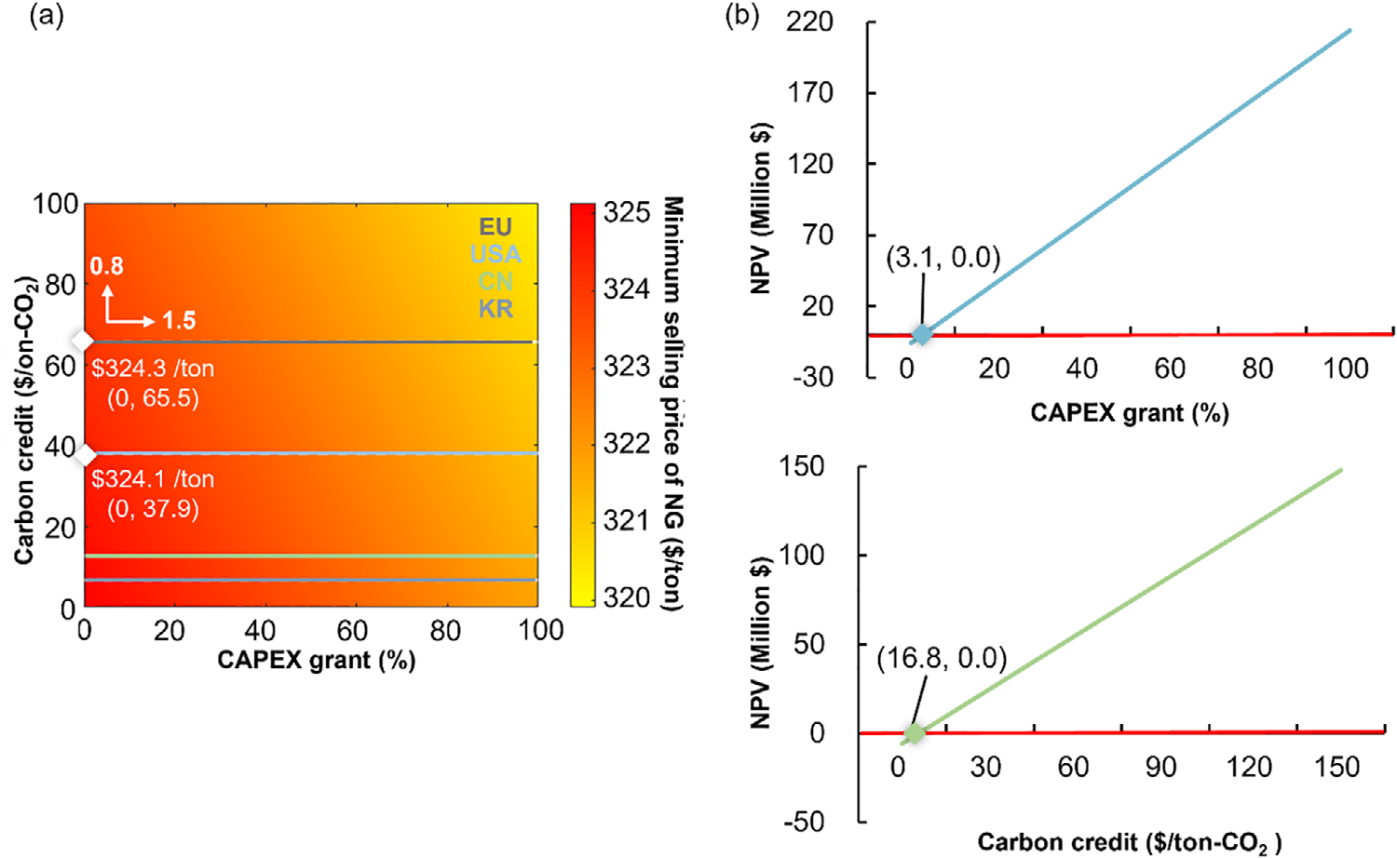
(a) Sensitivity of the eMSP of NG to carbon credit and CAPEX grant levels. (b) Net present value response to carbon credit and CAPEX grant levels.

The results indicate that the eMSP of NG decreases more rapidly with increasing CAPEX grant percentage than with increasing carbon credit levels. While higher carbon credit prices contribute to a reduction in eMSP by monetizing the environmental benefits associated with lower GWP, their impact remains comparatively moderate across the examined range. In contrast, CAPEX support directly lowers the annualized capital burden of the integrated system, leading to a steeper and more pronounced decrease in eMSP.

The contour lines further reveal that, for a given carbon credit level, incremental CAPEX grants yield substantial reductions in MSP, whereas equivalent increases in carbon credit prices result in smaller marginal benefits. This behavior reflects the underlying cost structure of the system, in which capital investment plays a more significant role than operational emissions costs in determining NG MSP.

Importantly, the analysis identifies distinct regions in the policy space where the eMSP of the ORC–PEMWE configuration falls below that of the conventional ORV system. These regions emerge under combinations of moderate carbon pricing and sufficiently high CAPEX support. As shown in Figure 9b, the NPV of ORC–PEMWE reaches the break‐even point when a CAPEX grant of approximately 3.1% is applied, even in the absence of carbon credits, or alternatively when the carbon credit price reaches about 16.8$/ton‐CO_2_ without CAPEX support.

Overall, the combined interpretation of Figure 9a,b demonstrates that ORC–PEMWE provides a pathway through which environmental benefits—specifically lower life cycle GWP—can be translated into tangible economic gains under appropriately designed policy frameworks. When supported by coordinated policy instruments, such as CAPEX grants and carbon credits, the functional expansion toward hydrogen production not only enhances system versatility but also enables the integrated configuration to achieve price competitiveness relative to conventional NG regasification.

Rather than representing a marginal or symbolic addition, hydrogen production becomes an economically meaningful function when policy support is aligned with the capital‐intensive nature of integrated LNG energy systems, allowing environmentally optimized configurations to compete directly with incumbent NG‐based systems.

## 4 Conclusions

This study redefines the potential of LNG regasification terminals, moving beyond simple gas supply to establish integrated energy hubs capable of co‐producing electricity and hydrogen. By conducting a system‐level evaluation of two parallel ORC loops coupled with optional hydrogen pathways (PEMWE and SWDE), this work elucidates the techno‐economic and environmental viability of functional expansion.

From a techno‐economic perspective, the ORC and ORC–PEMWE configurations maintained NG MSPs close to that of conventional regasification, with values of 323.2 and 324.6$/ton‐NG, respectively, compared with 324.5$/ton‐NG for ORV. In contrast, ORC–SWDE exhibited a higher MSP of 334.6$/ton‐NG under the current replacement interval and capital cost assumptions. The limited differences among ORV, ORC, and ORC–PEMWE are primarily attributable to the dominant contribution of LNG feedstock costs within the overall system.

Regarding hydrogen production, the ORC–PEMWE configuration emerged as the most balanced pathway, where byproduct revenues successfully offset additional capital requirements. In contrast, the ORC–SWDE configuration currently faces challenges associated with the conservative replacement interval, the resulting replacement‐related operating costs, the higher assumed CAPEX, and the limited technological maturity of direct seawater electrolysis. Environmentally, the integration proved highly effective; the ORC system reduced reliance on grid electricity, and the ORC–PEMWE pathway achieved a low GWP of 0.420 kg CO_2_‐eq/kg‐H_2_, satisfying stringent low‐carbon hydrogen standards without inducing significant trade‐offs.

The extended eTEA analysis provides critical policy insights. It demonstrates that while carbon credits alone offer limited leverage under a feedstock‐dominated cost structure, CAPEX‐oriented policy support is the key driver for economic parity. This underscores the necessity of targeted policy design to translate environmental benefits into tangible economic competitiveness.

In conclusion, this work shifts the paradigm of LNG cold energy utilization from a narrow efficiency problem to a broad strategy of functional expansion. The findings validate that LNG terminals can evolve into multi‐functional energy systems supplying natural gas, low‐carbon electricity, and hydrogen. This perspective offers a realistic, policy‐relevant framework for decarbonizing existing infrastructure while maintaining economic feasibility.

## Author Contributions

**Jiyoung Lee**: conceptualization, investigation, writing – original draft, writing – review and editing, methodology, visualization. **Byeongchan Ahn**: writing – review and editing, methodology, investigation. **Hyuntae Sohn**: funding acquisition, investigation, writing – review and editing. **Yeonsoo Kim**: funding acquisition, investigation, writing – review and editing. **Kiho Park**: investigation, writing – review and editing, visualization, methodology, conceptualization. **Wangyun Won**: funding acquisition, methodology, supervision, conceptualization, visualization, project administration, writing – review and editing.

## Conflicts of Interest

The authors declare no conflicts of interest.

## Supporting information




**Supporting File**: gch270166‐sup‐0001‐SuppMat.docx.

## Data Availability

The data that support the findings of this study are available from the corresponding author upon reasonable request.
