# Supplementary material for: System‐Level Assessment of LNG Cold Energy Utilization for Functional Expansion of Regasification Terminals
Source: Glob Chall. 2026 Oct 27;10(10):e70166. doi: 10.1002/gch2.70166 (PMC13711101; doi:10.1002/gch2.70166)
Supplement: Supplementary file 1 [file GCH2-10-e70166-s001.docx]

**System-level assessment of LNG cold energy utilization for functional expansion of regasification terminals**

**Jiyoung Lee^1^, Byeongchan Ahn^1^, Hyuntae Sohn^2^, Yeonsoo Kim^3^, Kiho Park^4*^_,_ Wangyun Won^1*^**

| **^1^** | Department of Chemical and Biological Engineering, Korea University, 145 Anam-ro,  Seongbuk-gu, Seoul 02841, Republic of Korea |
| --- | --- |
| ^2^ | Center for Hydrogen and Fuel Cell Research, Korea Institute of Science and Technology (KIST), 5 Hwarang-ro 14-gil, Seongbuk-gu, Seoul 02792, Republic of Korea |
| ^3^ | Department of Chemical Engineering, Kwangwoon University, 20 Kwangwoon-ro, Nowon-gu, Seoul 01897, Republic of Korea |
| ^4^ | Department of Chemical Engineering, Hanyang University, 222 Wangsimni-ro, Seongdong-gu, Seoul 04763, Republic of Korea |
| ^*^ | Corresponding authors (E-mail: kiho138@hanyang.ac.kr (K. Park) wwon@korea.ac.kr (W. Won)) |

**Supplementary Material**

**A. Working fluid screening and operating conditions**

The working fluid assessment was conducted in two stages. First, R170, R1270, R290, and R600a were screened using their saturation temperatures at the fixed turbine outlet pressure of 1.1 bar. The saturation temperatures were calculated using the Antoine equation parameters reported in the NIST Chemistry WebBook [1].

The preliminary comparison was used to classify the candidate fluids according to their relative phase change temperature levels. R170 represented the lowest temperature level, R1270 and R290 represented closely grouped intermediate temperature levels, and R600a represented a separate higher temperature level. R170 was assigned to the first ORC loop. R1270 and R290 were shortlisted as candidates for the second ORC loop, and R290 was retained as the representative working fluid for the selected ORC configuration. This screening was not intended as a comparative cycle performance optimization among the four candidate fluids.

After R170 and R290 were assigned to the two loops, their pump discharge pressures and working fluid mass flow rates were determined through Aspen Plus sensitivity analyses. The condenser outlet vapor fraction was fixed at zero for both loops to ensure complete condensation.

Table A.1. Saturation characteristics and preliminary classification of the candidate working fluids.

| Working fluid | Estimated saturation temperature at 1.1 bar (℃) | Critical temperature (℃) | Critical pressure (bar) | Relative phase change region |
| --- | --- | --- | --- | --- |
| R170 | -87.1 | 32.2 | 48.7 | Lowest temperature |
| R1270 | -45.9 | 91.7 | 46.0 | Intermediate, lower |
| R290 | -40.3 | 96.7 | 42.5 | Intermediate, higher |
| R600a | -9.6 | 152.0 | 38.0 | Highest temperature |

Table A.2. Selected operating conditions of the R170 and R290 ORC loops.

| Parmeter | R170 examined range | R170 selected value | R290 examined range | R290 selected value |
| --- | --- | --- | --- | --- |
| Pump discharge pressure (bar) | 5-25 | 20 | 1.2-10 | 7 |
| Working fluid mass flow rate (kg/s) | 20-25 | 24 | 30-35 | 30.8 |
| Turbine outlet pressure (bar) | Fixed | 1.1 | Fixed | 1.1 |
| Condenser outlet vapor fraction | Fixed | 0 | Fixed | 0 |

**B. Techno-economic analysis (TEA)**

The purchase and installed costs of the LNG regasification and ORC equipment were estimated directly using Aspen Economic Analyzer based on the equipment sizes obtained from the process simulation. Therefore, no user-specified capacity-scaling exponent was applied to these sections.

The desalination unit cost was estimated using the capacity-based cost data reported by the World Bank [2] and was scaled linearly with the required water production capacity. The PEMWE cost was estimated using the DOE [3] cost reported on a power-capacity basis and was scaled linearly with the required electrolyzer capacity. A scaling exponent of 1.0 was applied to both the desalination and PEMWE units.

The SWDE capital cost was represented using a multiplier of 2.0 relative to the PEMWE capital cost at an equivalent power capacity. This factor was treated as an exploratory scenario assumption rather than as a capacity-scaling exponent or a commercially established SWDE cost estimate.

All literature-based cost data were converted to a 2024 $ basis using the CEPCI values listed in Table B.1.

Table B.1. Major economic parameters and operating assumptions used in the TEA.

| Annual operating time (h/yr) ^a^ | | | 7,884 | |
| --- | --- | --- | --- | --- |
| Working fluid replacement rate (%/yr) | | | 2.0 | |
| PEMWE replacement interval (h) ^b^ | | | 40,000 | |
| SWDE assumed replacement interval (h) ^c^ | | | 500 | |
| LNG price ($/ton) ^d^ | | | 274 | |
| R170 price ($/ton) ^e^ | | | 140 | |
| R290 price ($/ton) ^f^ | | | 242 | |
| Electricity price (cent/kWh) | | | 8.06 | |
| Hydrogen selling price ($/ton) ^b^ | | | 7,500 | |
| CEPCI, 2016/2020/2022/2024 ^g^ | | | 542/596/816/799 | |
| Desalination unit operating cost ($/yr) ^h^ | | | 28,105 | |
| PEMWE unit operating cost ($/yr) ^b^ | | | 1,793,701 | |
| SWDE unit operating cost ($/yr) ^i^ | | | 143,677,305 | |
| PEMWE specific energy consumption (kWh/kg-H_2_) ^b^ | | | 57 | |
| SWDE specific energy consumption (kWh/kg-H_2_) ^c^ | | | 56 | |
| ^a^ | Taken from Davis et al. [4]. |  | |  |
| ^b^ | Taken from the U.S. Department of Energy [3]. |  | |  |
| ^c^ | Taken from Liu et al. [5]. |  | |  |
| ^d^ | Taken from Energy Information Administration [6]. |  | |  |
| ^e^ | Taken from Trading Economics [7]. |  | |  |
| ^f^ | Taken from Trading Economics [8]. |  | |  |
| ^g^ | Taken from Bailey [9] |  | |  |
| ^h^ | Taken from the World Bank [2]. |  | |  |
| ^i^ | Calculated using the 80-fold replacement-frequency ratio relative to PEMWE. |  | |  |

Table B.2. Cost worksheet of ORV.

| Subsystems | |  | | | Installed cost | |
| --- | --- | --- | --- | --- | --- | --- |
| LNG regasification | Regasification (ORV) | | | | $ | 35,119,800 |
|  | Storage | | | | $ | 2,385,083,478 |
| **Total installed equipment cost** | |  |  | | **$** | **2,420,203,278** |
| Warehouse | | 4.0% | | of ISBL^*^ | $ | 96,808,131 |
| Site development | | 9.0% | | of ISBL^*^ | $ | 217,818,295 |
| Additional piping | | 4.5% | | of ISBL^*^ | $ | 108,909,147 |
| **Total direct cost (TDC)** | |  | |  | $ | **2,843,738,851** |
| Prorateable expenses | | 10.0% | | of TDC | $ | 284,373,885 |
| Field expenses | | 10.0% | | of TDC | $ | 284,373,885 |
| Home office & construction fee | | 20.0% | | of TDC | $ | 568,747,770 |
| Project contingency | | 10.0% | | of TDC | $ | 284,373,885 |
| Other costs (start-up, permits, etc.) | | 10.0% | | of TDC | $ | 284,373,885 |
| **Total indirect cost** | |  | |  | **$** | **1,706,243,311** |
| **Fixed capital investment (FCI)** | |  | |  | **$** | **4,549,982,162** |
| Land | | 2.0% | | of FCI | $ | 90,999,643 |
| Working capital | | 5.0% | | of FCI | $ | 227,499,108 |
| **Total capital investment (TCI)** | |  | |  | **$** | **4,868,480,913** |
| ^*^ Inside battery limits (ISBL) include LNG regasification. | | | | | | |

Table B.3. Cost worksheet of ORC.

| Subsystems | |  | | | Installed cost | |
| --- | --- | --- | --- | --- | --- | --- |
| LNG regasification | Regasification (ORC) | | | | $ | 101,479,292 |
|  | Storage | | | | $ | 2,385,083,478 |
| **Total installed equipment cost** | |  |  | | **$** | **2,486,562,769** |
| Warehouse | | 4.0% | | of ISBL^*^ | $ | 99,462,511 |
| Site development | | 9.0% | | of ISBL^*^ | $ | 223,790,649 |
| Additional piping | | 4.5% | | of ISBL^*^ | $ | 111,895,325 |
| **Total direct cost (TDC)** | |  | |  | $ | **2,921,711,254** |
| Prorateable expenses | | 10.0% | | of TDC | $ | 292,171,125 |
| Field expenses | | 10.0% | | of TDC | $ | 292,171,125 |
| Home office & construction fee | | 20.0% | | of TDC | $ | 584,342,251 |
| Project contingency | | 10.0% | | of TDC | $ | 292,171,125 |
| Other costs (start-up, permits, etc.) | | 10.0% | | of TDC | $ | 292,171,125 |
| **Total indirect cost** | |  | |  | **$** | **1,753,026,752** |
| **Fixed capital investment (FCI)** | |  | |  | **$** | **4,674,738,006** |
| Land | | 2.0% | | of FCI | $ | 93,494,760 |
| Working capital | | 5.0% | | of FCI | $ | 233,736,900 |
| Refrigerant | |  | |  | $ | 626,128 |
| **Total capital investment (TCI)** | |  | |  | **$** | **5,002,595,795** |
| ^*^ Inside battery limits (ISBL) include LNG regasification. | | | | | | |

Table B.4. Cost worksheet of ORC-PEMWE.

| Subsystems | |  | | | Installed cost | |
| --- | --- | --- | --- | --- | --- | --- |
| LNG regasification | Regasification (ORC) | | | | $ | 101,479,292 |
|  | Storage | | | | $ | 2,385,083,478 |
| Hydrogen production | Desalination | | | | $ | 84,518 |
|  | PEMWE | | | | $ | 87,440,376 |
| **Total installed equipment cost** | |  |  | | **$** | **2,574,087,663** |
| Warehouse | | 4.0% | | of ISBL^*^ | $ | 102,963,507 |
| Site development | | 9.0% | | of ISBL^*^ | $ | 231,667,890 |
| Additional piping | | 4.5% | | of ISBL^*^ | $ | 115,833,945 |
| **Total direct cost (TDC)** | |  | |  | $ | **3,024,553,004** |
| Prorateable expenses | | 10.0% | | of TDC | $ | 302,455,300 |
| Field expenses | | 10.0% | | of TDC | $ | 302,455,300 |
| Home office & construction fee | | 20.0% | | of TDC | $ | 604,910,601 |
| Project contingency | | 10.0% | | of TDC | $ | 302,455,300 |
| Other costs (start-up, permits, etc.) | | 10.0% | | of TDC | $ | 302,455,300 |
| **Total indirect cost** | |  | |  | **$** | **1,814,731,802** |
| **Fixed capital investment (FCI)** | |  | |  | **$** | **4,839,284,806** |
| Land | | 2.0% | | of FCI | $ | 96,785,696 |
| Working capital | | 5.0% | | of FCI | $ | 241,964,240 |
| Refrigerant | |  | |  | $ | 626,128 |
| **Total capital investment (TCI)** | |  | |  | **$** | **5,178,660,871** |
| ^*^ Inside battery limits (ISBL) include LNG regasification, Desalination, and PEMWE. | | | | | | |

Table B.5. Cost worksheet of ORC-SWDE.

| Subsystems | |  | | | Installed cost | |
| --- | --- | --- | --- | --- | --- | --- |
| LNG regasification | Regasification (ORC) | | | | $ | 101,479,292 |
|  | Storage | | | | $ | 2,385,083,478 |
| Hydrogen production | SWDE | | | | $ | 175,101,653 |
| **Total installed equipment cost** | |  |  | | **$** | **2,661,664,423** |
| Warehouse | | 4.0% | | of ISBL^*^ | $ | 106,466,577 |
| Site development | | 9.0% | | of ISBL^*^ | $ | 239,549,798 |
| Additional piping | | 4.5% | | of ISBL^*^ | $ | 119,774,899 |
| **Total direct cost (TDC)** | |  | |  | $ | **3,127,455,697** |
| Prorateable expenses | | 10.0% | | of TDC | $ | 312,745,570 |
| Field expenses | | 10.0% | | of TDC | $ | 312,745,570 |
| Home office & construction fee | | 20.0% | | of TDC | $ | 625,491,139 |
| Project contingency | | 10.0% | | of TDC | $ | 312,745,570 |
| Other costs (start-up, permits, etc.) | | 10.0% | | of TDC | $ | 312,745,570 |
| **Total indirect cost** | |  | |  | **$** | **1,876,473,418** |
| **Fixed capital investment (FCI)** | |  | |  | **$** | **5,003,929,115** |
| Land | | 2.0% | | of FCI | $ | 100,078,582 |
| Working capital | | 5.0% | | of FCI | $ | 250,196,456 |
| Refrigerant | |  | |  | $ | 626,128 |
| **Total capital investment (TCI)** | |  | |  | **$** | **5,354,830,281** |
| ^*^ Inside battery limits (ISBL) include LNG regasification and the SWDE system. | | | | | | |

Table B.6. Discounted cash flow calculations of ORV.

| Year | | -2 | -1 | 0 | 1 | 2 | 3 | 4 | 5 |
| --- | --- | --- | --- | --- | --- | --- | --- | --- | --- |
| Fixed capital investment | | 145,599,429 | 1,091,995,719 | 582,397,717 |  |  |  |  |  |
| Land | | 90,999,643 |  |  |  |  |  |  |  |
| Working capital | |  |  | 227,499,108 |  |  |  |  |  |
| Loan payment | |  |  |  | 406,848,909 | 406,848,909 | 406,848,909 | 406,848,909 | 406,848,909 |
|  | Loan interest payment | 17,471,932 | 148,511,418 | 218,399,144 | 218,399,144 | 203,323,163 | 187,041,103 | 169,456,478 | 150,465,084 |
|  | Loan principal | 218,399,144 | 1,856,392,722 | 2,729,989,297 | 2,541,539,532 | 2,338,013,785 | 2,118,205,979 | 1,880,813,548 | 1,624,429,723 |
|  | NG sales |  |  |  | 4,144,804,321 | 5,526,405,761 | 5,526,405,761 | 5,526,405,761 | 5,526,405,761 |
| Total annual sales | | |  |  | 4,144,804,321 | 5,526,405,761 | 5,526,405,761 | 5,526,405,761 | 5,526,405,761 |
| Annual manufacturing cost | | |  |  |  |  |  |  |  |
|  | Feedstock |  |  |  | 4,085,212,229 | 4,668,813,976 | 4,668,813,976 | 4,668,813,976 | 4,668,813,976 |
|  | Other variable costs | |  |  | 7,127,599 | 8,145,828 | 8,145,828 | 8,145,828 | 8,145,828 |
|  | Fixed operating costs | |  |  | 172,705,706 | 172,705,706 | 172,705,706 | 172,705,706 | 172,705,706 |
| Total product cost | | |  |  | 4,265,045,534 | 4,849,665,510 | 4,849,665,510 | 4,849,665,510 | 4,849,665,510 |
| Annual depreciation | | |  |  |  |  |  |  |  |
| General plant writedown | | |  |  | 14.29% | 24.49% | 17.49% | 12.49% | 8.93% |
|  | Depreciation charge | |  |  | 650,192,451 | 1,114,290,631 | 795,791,880 | 568,292,772 | 406,313,407 |
|  | Remaining value | |  |  | 3,899,789,711 | 2,785,499,079 | 1,989,707,199 | 1,421,414,427 | 1,015,101,020 |
| Net revenue | |  |  |  | -988,832,808 | -640,873,543 | -306,092,732 | -61,008,999 | 119,961,760 |
| Losses forward | |  |  |  |  | -988,832,808 | -1,629,706,351 | -1,935,799,083 | -1,996,808,082 |
| Taxable income | |  |  |  | -988,832,808 | -1,629,706,351 | -1,935,799,083 | -1,996,808,082 | -1,876,846,322 |
| Income tax | |  |  |  | 0 | 0 | 0 | 0 | 0 |
| Annual cash income | | |  |  | -527,090,123 | 269,891,342 | 269,891,342 | 269,891,342 | 269,891,342 |
| Discount factor | | 1.210 | 1.100 | 1.000 | 0.909 | 0.826 | 0.751 | 0.683 | 0.621 |
| Annual present value | | |  |  | -479,172,839 | 223,050,696 | 202,773,360 | 184,339,418 | 167,581,289 |
| Total capital investment + interest | | 307,425,915 | 1,364,557,850 | 1,028,295,969 |  |  |  |  |  |
| Net present worth | | |  | 0 |  |  |  |  |  |

Table B.6. Discounted cash flow calculations of ORV (continued).

| Year | | 6 | 7 | 8 | 9 | 10 | 11 | 12 | 13 |
| --- | --- | --- | --- | --- | --- | --- | --- | --- | --- |
| Fixed capital investment | | |  |  |  |  |  |  |  |
| Land | |  |  |  |  |  |  |  |  |
| Working capital | |  |  |  |  |  |  |  |  |
| Loan payment | | 406,848,909 | 406,848,909 | 406,848,909 | 406,848,909 | 406,848,909 | 0 | 0 | 0 |
|  | Loan interest payment | 129,954,378 | 107,802,815 | 83,879,128 | 58,041,545 | 30,136,956 | 0 | 0 | 0 |
|  | Loan principal | 1,347,535,192 | 1,048,489,098 | 725,519,317 | 376,711,953 | 0 | 0 | 0 | 0 |
|  | NG sales | 5,526,405,761 | 5,526,405,761 | 5,526,405,761 | 5,526,405,761 | 5,526,405,761 | 5,526,405,761 | 5,526,405,761 | 5,526,405,761 |
| Total annual sales | | 5,526,405,761 | 5,526,405,761 | 5,526,405,761 | 5,526,405,761 | 5,526,405,761 | 5,526,405,761 | 5,526,405,761 | 5,526,405,761 |
| Annual manufacturing cost | | |  |  |  |  |  |  |  |
|  | Feedstock | 4,668,813,976 | 4,668,813,976 | 4,668,813,976 | 4,668,813,976 | 4,668,813,976 | 4,668,813,976 | 4,668,813,976 | 4,668,813,976 |
|  | Other variable costs | 8,145,828 | 8,145,828 | 8,145,828 | 8,145,828 | 8,145,828 | 8,145,828 | 8,145,828 | 8,145,828 |
|  | Fixed operating costs | 172,705,706 | 172,705,706 | 172,705,706 | 172,705,706 | 172,705,706 | 172,705,706 | 172,705,706 | 172,705,706 |
| Total product cost | | 4,849,665,510 | 4,849,665,510 | 4,849,665,510 | 4,849,665,510 | 4,849,665,510 | 4,849,665,510 | 4,849,665,510 | 4,849,665,510 |
| Annual depreciation | |  |  |  |  |  |  |  |  |
| General plant writedown | | 8.92% | 8.93% | 4.46% |  |  |  |  |  |
|  | Depreciation charge | 405,858,409 | 406,313,407 | 202,929,204 |  |  |  |  |  |
|  | Remaining value | 609,242,611 | 202,929,204 | 0 |  |  |  |  |  |
| Net revenue | | 140,927,465 | 162,624,029 | 389,931,919 | 618,698,706 | 646,603,295 | 676,740,251 | 676,740,251 | 676,740,251 |
| Losses forward | | -1,876,846,322 | -1,735,918,857 | -1,573,294,828 | -1,183,362,909 | -564,664,203 | 0 | 0 | 0 |
| Taxable income | | -1,735,918,857 | -1,573,294,828 | -1,183,362,909 | -564,664,203 | 81,939,092 | 676,740,251 | 676,740,251 | 676,740,251 |
| Income tax | | 0 | 0 | 0 | 0 | 17,207,209 | 142,115,453 | 142,115,453 | 142,115,453 |
| Annual cash income | | 269,891,342 | 269,891,342 | 269,891,342 | 269,891,342 | 252,684,133 | 534,624,798 | 534,624,798 | 534,624,798 |
| Discount factor | | 0.564 | 0.513 | 0.467 | 0.424 | 0.386 | 0.350 | 0.319 | 0.290 |
| Annual present value | | 152,346,627 | 138,496,933 | 125,906,303 | 114,460,275 | 97,420,672 | 187,382,730 | 170,347,937 | 154,861,761 |
| Total capital investment + interest | | |  |  |  |  |  |  |  |
| Net present worth | |  |  |  |  |  |  |  |  |

Table B.6. Discounted cash flow calculations of ORV (continued).

| Year | | 14 | 15 | 16 | 17 | 18 | 19 | 20 | 21 |
| --- | --- | --- | --- | --- | --- | --- | --- | --- | --- |
| Fixed capital investment | | |  |  |  |  |  |  |  |
| Land | |  |  |  |  |  |  |  |  |
| Working capital | |  |  |  |  |  |  |  |  |
| Loan payment | | 0 | 0 | 0 | 0 | 0 | 0 | 0 | 0 |
|  | Loan interest payment | 0 | 0 | 0 | 0 | 0 | 0 | 0 | 0 |
|  | Loan principal | 0 | 0 | 0 | 0 | 0 | 0 | 0 | 0 |
|  | NG sales | 5,526,405,761 | 5,526,405,761 | 5,526,405,761 | 5,526,405,761 | 5,526,405,761 | 5,526,405,761 | 5,526,405,761 | 5,526,405,761 |
| Total annual sales | | 5,526,405,761 | 5,526,405,761 | 5,526,405,761 | 5,526,405,761 | 5,526,405,761 | 5,526,405,761 | 5,526,405,761 | 5,526,405,761 |
| Annual manufacturing cost | | |  |  |  |  |  |  |  |
|  | Feedstock | 4,668,813,976 | 4,668,813,976 | 4,668,813,976 | 4,668,813,976 | 4,668,813,976 | 4,668,813,976 | 4,668,813,976 | 4,668,813,976 |
|  | Other variable costs | 8,145,828 | 8,145,828 | 8,145,828 | 8,145,828 | 8,145,828 | 8,145,828 | 8,145,828 | 8,145,828 |
|  | Fixed operating costs | 172,705,706 | 172,705,706 | 172,705,706 | 172,705,706 | 172,705,706 | 172,705,706 | 172,705,706 | 172,705,706 |
| Total product cost | | 4,849,665,510 | 4,849,665,510 | 4,849,665,510 | 4,849,665,510 | 4,849,665,510 | 4,849,665,510 | 4,849,665,510 | 4,849,665,510 |
| Annual depreciation | |  |  |  |  |  |  |  |  |
| General plant writedown | | |  |  |  |  |  |  |  |
|  | Depreciation charge | |  |  |  |  |  |  |  |
|  | Remaining value |  |  |  |  |  |  |  |  |
| Net revenue | | 676,740,251 | 676,740,251 | 676,740,251 | 676,740,251 | 676,740,251 | 676,740,251 | 676,740,251 | 676,740,251 |
| Losses forward | | 0 | 0 | 0 | 0 | 0 | 0 | 0 | 0 |
| Taxable income | | 676,740,251 | 676,740,251 | 676,740,251 | 676,740,251 | 676,740,251 | 676,740,251 | 676,740,251 | 676,740,251 |
| Income tax | | 142,115,453 | 142,115,453 | 142,115,453 | 142,115,453 | 142,115,453 | 142,115,453 | 142,115,453 | 142,115,453 |
| Annual cash income | | 534,624,798 | 534,624,798 | 534,624,798 | 534,624,798 | 534,624,798 | 534,624,798 | 534,624,798 | 534,624,798 |
| Discount factor | | 0.263 | 0.239 | 0.218 | 0.198 | 0.180 | 0.164 | 0.149 | 0.135 |
| Annual present value | | 140,783,419 | 127,984,926 | 116,349,933 | 105,772,666 | 96,156,969 | 87,415,427 | 79,468,570 | 72,244,154 |
| Total capital investment + interest | | |  |  |  |  |  |  |  |
| Net present worth | |  |  |  |  |  |  |  |  |

Table B.6. Discounted cash flow calculations of ORV (continued).

| Year | 22 | | | | 23 | 24 | 25 | 26 | 27 | 28 | 29 | 30 |
| --- | --- | --- | --- | --- | --- | --- | --- | --- | --- | --- | --- | --- |
| Fixed capital investment | | | | | |  |  |  |  |  |  |  |
| Land | | | | |  |  |  |  |  |  |  | -90,999,643 |
| Working capital | | | | |  |  |  |  |  |  |  | -227,499,108 |
| Loan payment | | | | 0 | 0 | 0 | 0 | 0 | 0 | 0 | 0 | 0 |
| Loan interest | | | | 0 | 0 | 0 | 0 | 0 | 0 | 0 | 0 | 0 |
| Loan principal | | | | 0 | 0 | 0 | 0 | 0 | 0 | 0 | 0 | 0 |
| NG sales | 5,526,405,761 | | | | 5,526,405,761 | 5,526,405,761 | 5,526,405,761 | 5,526,405,761 | 5,526,405,761 | 5,526,405,761 | 5,526,405,761 | 5,526,405,761 |
| Total annual sales | 5,526,405,761 | | | | 5,526,405,761 | 5,526,405,761 | 5,526,405,761 | 5,526,405,761 | 5,526,405,761 | 5,526,405,761 | 5,526,405,761 | 5,526,405,761 |
| Annual manufacturing cost | | | | | |  |  |  |  |  |  |  |
| Feedstock | | 4,668,813,976 | | | 4,668,813,976 | 4,668,813,976 | 4,668,813,976 | 4,668,813,976 | 4,668,813,976 | 4,668,813,976 | 4,668,813,976 | 4,668,813,976 |
|  | |  | | |  |  |  | 0 |  |  |  |  |
| Other variable costs | | 8,145,828 | | | 8,145,828 | 8,145,828 | 8,145,828 | 8,145,828 | 8,145,828 | 8,145,828 | 8,145,828 | 8,145,828 |
| Fixed operating costs | | 172,705,706 | | | 172,705,706 | 172,705,706 | 172,705,706 | 172,705,706 | 172,705,706 | 172,705,706 | 172,705,706 | 172,705,706 |
| Total product cost | 4,849,665,510 | | | | 4,849,665,510 | 4,849,665,510 | 4,849,665,510 | 4,849,665,510 | 4,849,665,510 | 4,849,665,510 | 4,849,665,510 | 4,849,665,510 |
| Annual depreciation | | | | |  |  |  |  |  |  |  |  |
| General plant writedown | | | | | |  |  |  |  |  |  |  |
| Depreciation charge | | |  | |  |  |  |  |  |  |  |  |
| Remaining value | | |  | |  |  |  |  |  |  |  |  |
| Net revenue | 676,740,251 | | | | 676,740,251 | 676,740,251 | 676,740,251 | 676,740,251 | 676,740,251 | 676,740,251 | 676,740,251 | 676,740,251 |
| Losses forward | 0 | | | | 0 | 0 | 0 | 0 | 0 | 0 | 0 | 0 |
| Taxable income | 676,740,251 | | | | 676,740,251 | 676,740,251 | 676,740,251 | 676,740,251 | 676,740,251 | 676,740,251 | 676,740,251 | 676,740,251 |
| Income tax | 142,115,453 | | | | 142,115,453 | 142,115,453 | 142,115,453 | 142,115,453 | 142,115,453 | 142,115,453 | 142,115,453 | 142,115,453 |
| Annual cash income | 534,624,798 | | | | 534,624,798 | 534,624,798 | 534,624,798 | 534,624,798 | 534,624,798 | 534,624,798 | 534,624,798 | 534,624,798 |
| Discount factor | 0.123 | | | | 0.112 | 0.102 | 0.092 | 0.084 | 0.076 | 0.069 | 0.063 | 0.057 |
| Annual present value | 65,676,504 | | | | 59,705,913 | 54,278,102 | 49,343,729 | 44,857,936 | 40,779,942 | 37,072,674 | 33,702,431 | 30,638,574 |
| Total capital investment + interest | | | | | |  |  |  |  |  |  | -18,252,703 |
| Net present worth | | | | |  |  |  |  |  |  |  |  |

Table B.7. Discounted cash flow calculations of ORC.

| Year | | -2 | -1 | 0 | 1 | 2 | 3 | 4 | 5 |
| --- | --- | --- | --- | --- | --- | --- | --- | --- | --- |
| Fixed capital investment | | 149,591,616 | 1,121,937,122 | 598,366,465 |  |  |  |  |  |
| Land | | 93,494,760 |  |  |  |  |  |  |  |
| Working fluid | |  |  | 626,128 |  |  |  |  |  |
| Working capital | |  |  | 233,736,900 |  |  |  |  |  |
| Loan payment | |  |  |  | 418,004,289 | 418,004,289 | 418,004,289 | 418,004,289 | 418,004,289 |
|  | Loan interest payment | 17,950,994 | 152,583,449 | 224,387,424 | 224,387,424 | 208,898,075 | 192,169,578 | 174,102,801 | 154,590,682 |
|  | Loan principal | 224,387,424 | 1,907,293,107 | 2,804,842,804 | 2,611,225,939 | 2,402,119,725 | 2,176,285,014 | 1,932,383,527 | 1,668,969,920 |
|  | Electricity sales | |  |  | 27,289,418 | 36,385,891 | 36,385,891 | 36,385,891 | 36,385,891 |
|  | NG sales | |  |  | 4,127,574,304 | 5,503,432,405 | 5,503,432,405 | 5,503,432,405 | 5,503,432,405 |
| Total annual sales | | |  |  | 4,154,863,722 | 5,539,818,297 | 5,539,818,297 | 5,539,818,297 | 5,539,818,297 |
| Annual manufacturing cost | | |  |  |  |  |  |  |  |
|  | Feedstock |  |  |  | 4,085,212,229 | 4,668,813,976 | 4,668,813,976 | 4,668,813,976 | 4,668,813,976 |
|  | Other variable costs | |  |  | 16,827 | 19,231 | 19,231 | 19,231 | 19,231 |
|  | Fixed operating costs | |  |  | 177,441,119 | 177,441,119 | 177,441,119 | 177,441,119 | 177,441,119 |
| Total product cost | | |  |  | 4,262,670,176 | 4,846,274,326 | 4,846,274,326 | 4,846,274,326 | 4,846,274,326 |
| Annual depreciation | | |  |  |  |  |  |  |  |
| General plant writedown | | |  |  | 14.29% | 24.49% | 17.49% | 12.49% | 8.93% |
|  | Depreciation charge | |  |  | 668,020,061 | 1,144,843,338 | 817,611,677 | 583,874,777 | 417,454,104 |
|  | Remaining value | |  |  | 4,006,717,945 | 2,861,874,607 | 2,044,262,930 | 1,460,388,153 | 1,042,934,049 |
| Net revenue | |  |  |  | -1,000,213,938 | -660,197,443 | -316,237,285 | -64,433,608 | 121,499,184 |
| Losses forward | |  |  |  |  | -1,000,213,938 | -1,660,411,381 | -1,976,648,666 | -2,041,082,274 |
| Taxable income | |  |  |  | -1,000,213,938 | -1,660,411,381 | -1,976,648,666 | -2,041,082,274 | -1,919,583,090 |
| Income tax | |  |  |  | 0 | 0 | 0 | 0 | 0 |
| Annual cash income | | |  |  | -525,810,742 | 275,539,681 | 275,539,681 | 275,539,681 | 275,539,681 |
| Discount factor | | 1.2100 | 1.1000 | 1.0000 | 0.9091 | 0.8264 | 0.7513 | 0.6830 | 0.6209 |
| Annual present value | | |  |  | -478,009,765 | 227,718,745 | 207,017,041 | 188,197,310 | 171,088,463 |
| Total capital investment + interest | | 315,855,218 | 1,401,972,627 | 1,057,116,918 |  |  |  |  |  |
| Net present worth | | |  | 0 |  |  |  |  |  |

Table B.7. Discounted cash flow calculations of ORC (continued).

| Year | | 6 | 7 | 8 | 9 | 10 | 11 | 12 | 13 |
| --- | --- | --- | --- | --- | --- | --- | --- | --- | --- |
| Fixed capital investment | | |  |  |  |  |  |  |  |
| Land | |  |  |  |  |  |  |  |  |
| Working fluid | |  |  |  |  |  |  |  |  |
| Working capital | |  |  |  |  |  |  |  |  |
| Loan payment | | 418,004,289 | 418,004,289 | 418,004,289 | 418,004,289 | 418,004,289 | 0 | 0 | 0 |
|  | Loan interest payment | 133,517,594 | 110,758,658 | 86,179,007 | 59,632,985 | 30,963,281 | 0 | 0 | 0 |
|  | Loan principal | 1,384,483,225 | 1,077,237,594 | 745,412,312 | 387,041,008 | 0 | 0 | 0 | 0 |
|  | Electricity sales | 36,385,891 | 36,385,891 | 36,385,891 | 36,385,891 | 36,385,891 | 36,385,891 | 36,385,891 | 36,385,891 |
|  | NG sales | 5,503,432,405 | 5,503,432,405 | 5,503,432,405 | 5,503,432,405 | 5,503,432,405 | 5,503,432,405 | 5,503,432,405 | 5,503,432,405 |
| Total annual sales | | 5,539,818,297 | 5,539,818,297 | 5,539,818,297 | 5,539,818,297 | 5,539,818,297 | 5,539,818,297 | 5,539,818,297 | 5,539,818,297 |
| Annual manufacturing cost | | |  |  |  |  |  |  |  |
|  | Feedstock | 4,668,813,976 | 4,668,813,976 | 4,668,813,976 | 4,668,813,976 | 4,668,813,976 | 4,668,813,976 | 4,668,813,976 | 4,668,813,976 |
|  | Other variable costs | 19,231 | 19,231 | 19,231 | 19,231 | 19,231 | 19,231 | 19,231 | 19,231 |
|  | Fixed operating costs | 177,441,119 | 177,441,119 | 177,441,119 | 177,441,119 | 177,441,119 | 177,441,119 | 177,441,119 | 177,441,119 |
| Total product cost | | 4,846,274,326 | 4,846,274,326 | 4,846,274,326 | 4,846,274,326 | 4,846,274,326 | 4,846,274,326 | 4,846,274,326 | 4,846,274,326 |
| Annual depreciation | |  |  |  |  |  |  |  |  |
| General plant writedown | | 8.92% | 8.93% | 4.46% |  |  |  |  |  |
|  | Depreciation charge | 416,986,630 | 417,454,104 | 208,493,315 |  |  |  |  |  |
|  | Remaining value | 625,947,419 | 208,493,315 | 0 |  |  |  |  |  |
| Net revenue | | 143,039,746 | 165,331,208 | 398,871,648 | 633,910,985 | 662,580,690 | 693,543,970 | 693,543,970 | 693,543,970 |
| Losses forward | | -1,919,583,090 | -1,776,543,344 | -1,611,212,135 | -1,212,340,488 | -578,429,502 | 0 | 0 | 0 |
| Taxable income | | -1,776,543,344 | -1,611,212,135 | -1,212,340,488 | -578,429,502 | 84,151,187 | 693,543,970 | 693,543,970 | 693,543,970 |
| Income tax | | 0 | 0 | 0 | 0 | 17,671,749 | 145,644,234 | 145,644,234 | 145,644,234 |
| Annual cash income | | 275,539,681 | 275,539,681 | 275,539,681 | 275,539,681 | 257,867,932 | 547,899,736 | 547,899,736 | 547,899,736 |
| Discount factor | | 0.5645 | 0.5132 | 0.4665 | 0.4241 | 0.3855 | 0.3505 | 0.3186 | 0.2897 |
| Annual present value | | 155,534,967 | 141,395,424 | 128,541,295 | 116,855,723 | 99,419,251 | 192,035,515 | 174,577,741 | 158,707,037 |
| Total capital investment + interest | | |  |  |  |  |  |  |  |
| Net present worth | |  |  |  |  |  |  |  |  |

Table B.7. Discounted cash flow calculations of ORC (continued).

| Year | | 14 | 15 | 16 | 17 | 18 | 19 | 20 | 21 |
| --- | --- | --- | --- | --- | --- | --- | --- | --- | --- |
| Fixed capital investment | | |  |  |  |  |  |  |  |
| Land | |  |  |  |  |  |  |  |  |
| Working fluid | |  |  |  |  |  |  |  |  |
| Working capital | |  |  |  |  |  |  |  |  |
| Loan payment | | 0 | 0 | 0 | 0 | 0 | 0 | 0 | 0 |
|  | Loan interest payment | 0 | 0 | 0 | 0 | 0 | 0 | 0 | 0 |
|  | Loan principal | 0 | 0 | 0 | 0 | 0 | 0 | 0 | 0 |
|  | Electricity sales | 36,385,891 | 36,385,891 | 36,385,891 | 36,385,891 | 36,385,891 | 36,385,891 | 36,385,891 | 36,385,891 |
|  | NG sales | 5,503,432,405 | 5,503,432,405 | 5,503,432,405 | 5,503,432,405 | 5,503,432,405 | 5,503,432,405 | 5,503,432,405 | 5,503,432,405 |
| Total annual sales | | 5,539,818,297 | 5,539,818,297 | 5,539,818,297 | 5,539,818,297 | 5,539,818,297 | 5,539,818,297 | 5,539,818,297 | 5,539,818,297 |
| Annual manufacturing cost | | |  |  |  |  |  |  |  |
|  | Feedstock | 4,668,813,976 | 4,668,813,976 | 4,668,813,976 | 4,668,813,976 | 4,668,813,976 | 4,668,813,976 | 4,668,813,976 | 4,668,813,976 |
|  | Other variable costs | 19,231 | 19,231 | 19,231 | 19,231 | 19,231 | 19,231 | 19,231 | 19,231 |
|  | Fixed operating costs | 177,441,119 | 177,441,119 | 177,441,119 | 177,441,119 | 177,441,119 | 177,441,119 | 177,441,119 | 177,441,119 |
| Total product cost | | 4,846,274,326 | 4,846,274,326 | 4,846,274,326 | 4,846,274,326 | 4,846,274,326 | 4,846,274,326 | 4,846,274,326 | 4,846,274,326 |
| Annual depreciation | |  |  |  |  |  |  |  |  |
| General plant writedown | | |  |  |  |  |  |  |  |
|  | Depreciation charge | |  |  |  |  |  |  |  |
|  | Remaining value |  |  |  |  |  |  |  |  |
| Net revenue | | 693,543,970 | 693,543,970 | 693,543,970 | 693,543,970 | 693,543,970 | 693,543,970 | 693,543,970 | 693,543,970 |
| Losses forward | | 0 | 0 | 0 | 0 | 0 | 0 | 0 | 0 |
| Taxable income | | 693,543,970 | 693,543,970 | 693,543,970 | 693,543,970 | 693,543,970 | 693,543,970 | 693,543,970 | 693,543,970 |
| Income tax | | 145,644,234 | 145,644,234 | 145,644,234 | 145,644,234 | 145,644,234 | 145,644,234 | 145,644,234 | 145,644,234 |
| Annual cash income | | 547,899,736 | 547,899,736 | 547,899,736 | 547,899,736 | 547,899,736 | 547,899,736 | 547,899,736 | 547,899,736 |
| Discount factor | | 0.2633 | 0.2394 | 0.2176 | 0.1978 | 0.1799 | 0.1635 | 0.1486 | 0.1351 |
| Annual present value | | 144,279,125 | 131,162,841 | 119,238,946 | 108,399,042 | 98,544,584 | 89,585,985 | 81,441,805 | 74,038,004 |
| Total capital investment + interest | | |  |  |  |  |  |  |  |
| Net present worth | |  |  |  |  |  |  |  |  |

Table B.7. Discounted cash flow calculations of ORC (continued).

| Year | | 22 | 23 | 24 | 25 | 26 | 27 | 28 | 29 | 30 |
| --- | --- | --- | --- | --- | --- | --- | --- | --- | --- | --- |
| Fixed capital investment | | |  |  |  |  |  |  |  |  |
| Land | |  |  |  |  |  |  |  |  | -93,494,760 |
| Working fluid | |  |  |  |  |  |  |  |  | -626,128 |
| Working capital | |  |  |  |  |  |  |  |  | -233,736,900 |
| Loan payment | | 0 | 0 | 0 | 0 | 0 | 0 | 0 | 0 | 0 |
|  | Loan interest payment | 0 | 0 | 0 | 0 | 0 | 0 | 0 | 0 | 0 |
|  | Loan principal | 0 | 0 | 0 | 0 | 0 | 0 | 0 | 0 | 0 |
|  | Electricity sales | 36,385,891 | 36,385,891 | 36,385,891 | 36,385,891 | 36,385,891 | 36,385,891 | 36,385,891 | 36,385,891 | 36,385,891 |
|  | NG sales | 5,503,432,405 | 5,503,432,405 | 5,503,432,405 | 5,503,432,405 | 5,503,432,405 | 5,503,432,405 | 5,503,432,405 | 5,503,432,405 | 5,503,432,405 |
| Total annual sales | | 5,539,818,297 | 5,539,818,297 | 5,539,818,297 | 5,539,818,297 | 5,539,818,297 | 5,539,818,297 | 5,539,818,297 | 5,539,818,297 | 5,539,818,297 |
| Annual manufacturing cost | | |  |  |  |  |  |  |  |  |
|  | Feedstock | 4,668,813,976 | 4,668,813,976 | 4,668,813,976 | 4,668,813,976 | 4,668,813,976 | 4,668,813,976 | 4,668,813,976 | 4,668,813,976 | 4,668,813,976 |
|  | Other variable costs | 19,231 | 19,231 | 19,231 | 19,231 | 19,231 | 19,231 | 19,231 | 19,231 | 19,231 |
|  | Fixed operating costs | 177,441,119 | 177,441,119 | 177,441,119 | 177,441,119 | 177,441,119 | 177,441,119 | 177,441,119 | 177,441,119 | 177,441,119 |
| Total product cost | | 4,846,274,326 | 4,846,274,326 | 4,846,274,326 | 4,846,274,326 | 4,846,274,326 | 4,846,274,326 | 4,846,274,326 | 4,846,274,326 | 4,846,274,326 |
| Annual depreciation | |  |  |  |  |  |  |  |  |  |
| General plant writedown | | |  |  |  |  |  |  |  |  |
|  | Depreciation charge | |  |  |  |  |  |  |  |  |
|  | Remaining value |  |  |  |  |  |  |  |  |  |
| Net revenue | | 693,543,970 | 693,543,970 | 693,543,970 | 693,543,970 | 693,543,970 | 693,543,970 | 693,543,970 | 693,543,970 | 693,543,970 |
| Losses forward | | 0 | 0 | 0 | 0 | 0 | 0 | 0 | 0 | 0 |
| Taxable income | | 693,543,970 | 693,543,970 | 693,543,970 | 693,543,970 | 693,543,970 | 693,543,970 | 693,543,970 | 693,543,970 | 693,543,970 |
| Income tax | | 145,644,234 | 145,644,234 | 145,644,234 | 145,644,234 | 145,644,234 | 145,644,234 | 145,644,234 | 145,644,234 | 145,644,234 |
| Annual cash income | | 547,899,736 | 547,899,736 | 547,899,736 | 547,899,736 | 547,899,736 | 547,899,736 | 547,899,736 | 547,899,736 | 547,899,736 |
| Discount factor | | 0.1228 | 0.1117 | 0.1015 | 0.0923 | 0.0839 | 0.0763 | 0.0693 | 0.0630 | 0.0573 |
| Annual present value | | 67,307,277 | 61,188,433 | 55,625,848 | 50,568,953 | 45,971,776 | 41,792,523 | 37,993,203 | 34,539,275 | 31,399,341 |
| Total capital investment + interest | | |  |  |  |  |  |  |  | -18,789,056 |
| Net present worth | |  |  |  |  |  |  |  |  |  |

Table B.8. Discounted cash flow calculations of ORC-PEMWE.

| Year | | -2 | -1 | 0 | 1 | 2 | 3 | 4 | 5 |
| --- | --- | --- | --- | --- | --- | --- | --- | --- | --- |
| Fixed capital investment | | 154,857,114 | 1,161,428,353 | 619,428,455 |  |  |  |  |  |
| Land | | 96,785,696 |  |  |  |  |  |  |  |
| Working fluid | |  |  | 626,128 |  |  |  |  |  |
| Working capital | |  |  | 241,964,240 |  |  |  |  |  |
| Loan payment | |  |  |  | 432,717,684 | 432,717,684 | 432,717,684 | 432,717,684 | 432,717,684 |
|  | Loan interest payment | 18,582,854 | 157,954,256 | 232,285,671 | 232,285,671 | 216,251,110 | 198,933,784 | 180,231,072 | 160,032,143 |
|  | Loan principal | 232,285,671 | 1,974,428,201 | 2,903,570,884 | 2,703,138,870 | 2,486,672,296 | 2,252,888,395 | 2,000,401,782 | 1,727,716,241 |
|  | Hydrogen sales | |  |  | 32,183,623 | 42,911,497 | 42,911,497 | 42,911,497 | 42,911,497 |
|  | NG sales |  |  |  | 4,145,372,612 | 5,527,163,482 | 5,527,163,482 | 5,527,163,482 | 5,527,163,482 |
| Total annual sales | | |  |  | 4,177,556,235 | 5,570,074,980 | 5,570,074,980 | 5,570,074,980 | 5,570,074,980 |
| Annual manufacturing cost | | |  |  |  |  |  |  |  |
|  | Feedstock |  |  |  | 4,085,212,229 | 4,668,813,976 | 4,668,813,976 | 4,668,813,976 | 4,668,813,976 |
|  | Other variable costs | |  |  | 1,571,083 | 1,795,524 | 1,795,524 | 1,795,524 | 1,795,524 |
|  | Fixed operating costs | |  |  | 183,686,896 | 183,686,896 | 183,686,896 | 183,686,896 | 183,686,896 |
| Total product cost | | |  |  | 4,270,470,208 | 4,854,296,396 | 4,854,296,396 | 4,854,296,396 | 4,854,296,396 |
| Annual depreciation | | |  |  |  |  |  |  |  |
| General plant writedown | | |  |  | 14.29% | 24.49% | 17.49% | 12.49% | 8.93% |
|  | Depreciation charge | |  |  | 691,533,799 | 1,185,140,849 | 846,390,913 | 604,426,672 | 432,148,133 |
|  | Remaining value | |  |  | 4,147,751,007 | 2,962,610,158 | 2,116,219,246 | 1,511,792,573 | 1,079,644,440 |
| Net revenue | |  |  |  | -1,016,733,443 | -685,613,375 | -329,546,112 | -68,879,160 | 123,598,308 |
| Losses forward | |  |  |  |  | -1,016,733,443 | -1,702,346,818 | -2,031,892,930 | -2,100,772,091 |
| Taxable income | |  |  |  | -1,016,733,443 | -1,702,346,818 | -2,031,892,930 | -2,100,772,091 | -1,977,173,783 |
| Income tax | |  |  |  | 0 | 0 | 0 | 0 | 0 |
| Annual cash income | | |  |  | -525,631,658 | 283,060,900 | 283,060,900 | 283,060,900 | 283,060,900 |
| Discount factor | | 1.2100 | 1.1000 | 1.0000 | 0.9091 | 0.8264 | 0.7513 | 0.6830 | 0.6209 |
| Annual present value | | |  |  | -477,846,962 | 233,934,628 | 212,667,843 | 193,334,403 | 175,758,548 |
| Total capital investment + interest | | 326,973,053 | 1,451,320,870 | 1,094,304,494 |  |  |  |  |  |
| Net present worth | | |  | 0 |  |  |  |  |  |

Table B.8. Discounted cash flow calculations of ORC-PEMWE (continued).

| Year | | 6 | 7 | 8 | 9 | 10 | 11 | 12 | 13 |
| --- | --- | --- | --- | --- | --- | --- | --- | --- | --- |
| Fixed capital investment | | |  |  |  |  |  |  |  |
| Land | |  |  |  |  |  |  |  |  |
| Working fluid | |  |  |  |  |  |  |  |  |
| Working capital | |  |  |  |  |  |  |  |  |
| Loan payment | | 432,717,684 | 432,717,684 | 432,717,684 | 432,717,684 | 432,717,684 | 0 | 0 | 0 |
|  | Loan interest payment | 138,217,299 | 114,657,268 | 89,212,435 | 61,732,015 | 32,053,162 | 0 | 0 | 0 |
|  | Loan principal | 1,433,215,856 | 1,115,155,440 | 771,650,191 | 400,664,522 | 0 | 0 | 0 | 0 |
|  | Hydrogen sales | 42,911,497 | 42,911,497 | 42,911,497 | 42,911,497 | 42,911,497 | 42,911,497 | 42,911,497 | 42,911,497 |
|  | NG sales | 5,527,163,482 | 5,527,163,482 | 5,527,163,482 | 5,527,163,482 | 5,527,163,482 | 5,527,163,482 | 5,527,163,482 | 5,527,163,482 |
| Total annual sales | | 5,570,074,980 | 5,570,074,980 | 5,570,074,980 | 5,570,074,980 | 5,570,074,980 | 5,570,074,980 | 5,570,074,980 | 5,570,074,980 |
| Annual manufacturing cost | | |  |  |  |  |  |  |  |
|  | Feedstock | 4,668,813,976 | 4,668,813,976 | 4,668,813,976 | 4,668,813,976 | 4,668,813,976 | 4,668,813,976 | 4,668,813,976 | 4,668,813,976 |
|  | Other variable costs | 1,795,524 | 1,795,524 | 1,795,524 | 1,795,524 | 1,795,524 | 1,795,524 | 1,795,524 | 1,795,524 |
|  | Fixed operating costs | 183,686,896 | 183,686,896 | 183,686,896 | 183,686,896 | 183,686,896 | 183,686,896 | 183,686,896 | 183,686,896 |
| Total product cost | | 4,854,296,396 | 4,854,296,396 | 4,854,296,396 | 4,854,296,396 | 4,854,296,396 | 4,854,296,396 | 4,854,296,396 | 4,854,296,396 |
| Annual depreciation | |  |  |  |  |  |  |  |  |
| General plant writedown | | 8.92% | 8.93% | 4.46% |  |  |  |  |  |
|  | Depreciation charge | 431,664,205 | 432,148,133 | 215,832,102 |  |  |  |  |  |
|  | Remaining value | 647,980,236 | 215,832,102 | 0 |  |  |  |  |  |
| Net revenue | | 145,897,080 | 168,973,182 | 410,734,046 | 654,046,568 | 683,725,422 | 715,778,584 | 715,778,584 | 715,778,584 |
| Losses forward | | -1,977,173,783 | -1,831,276,703 | -1,662,303,521 | -1,251,569,475 | -597,522,906 | 0 | 0 | 0 |
| Taxable income | | -1,831,276,703 | -1,662,303,521 | -1,251,569,475 | -597,522,906 | 86,202,516 | 715,778,584 | 715,778,584 | 715,778,584 |
| Income tax | | 0 | 0 | 0 | 0 | 18,102,528 | 150,313,503 | 150,313,503 | 150,313,503 |
| Annual cash income | | 283,060,900 | 283,060,900 | 283,060,900 | 283,060,900 | 264,958,371 | 565,465,081 | 565,465,081 | 565,465,081 |
| Discount factor | | 0.5645 | 0.5132 | 0.4665 | 0.4241 | 0.3855 | 0.3505 | 0.3186 | 0.2897 |
| Annual present value | | 159,780,498 | 145,254,999 | 132,049,999 | 120,045,453 | 102,152,922 | 198,192,061 | 180,174,601 | 163,795,092 |
| Total capital investment + interest | | |  |  |  |  |  |  |  |
| Net present worth | |  |  |  |  |  |  |  |  |

Table B.8. Discounted cash flow calculations of ORC-PEMWE (continued).

| Year | | 14 | 15 | 16 | 17 | 18 | 19 | 20 | 21 |
| --- | --- | --- | --- | --- | --- | --- | --- | --- | --- |
| Fixed capital investment | | |  |  |  |  |  |  |  |
| Land | |  |  |  |  |  |  |  |  |
| Working fluid | |  |  |  |  |  |  |  |  |
| Working capital | |  |  |  |  |  |  |  |  |
| Loan payment | | 0 | 0 | 0 | 0 | 0 | 0 | 0 | 0 |
|  | Loan interest payment | 0 | 0 | 0 | 0 | 0 | 0 | 0 | 0 |
|  | Loan principal | 0 | 0 | 0 | 0 | 0 | 0 | 0 | 0 |
|  | Hydrogen sales | 42,911,497 | 42,911,497 | 42,911,497 | 42,911,497 | 42,911,497 | 42,911,497 | 42,911,497 | 42,911,497 |
|  | NG sales | 5,527,163,482 | 5,527,163,482 | 5,527,163,482 | 5,527,163,482 | 5,527,163,482 | 5,527,163,482 | 5,527,163,482 | 5,527,163,482 |
| Total annual sales | | 5,570,074,980 | 5,570,074,980 | 5,570,074,980 | 5,570,074,980 | 5,570,074,980 | 5,570,074,980 | 5,570,074,980 | 5,570,074,980 |
| Annual manufacturing cost | | |  |  |  |  |  |  |  |
|  | Feedstock | 4,668,813,976 | 4,668,813,976 | 4,668,813,976 | 4,668,813,976 | 4,668,813,976 | 4,668,813,976 | 4,668,813,976 | 4,668,813,976 |
|  | Other variable costs | 1,795,524 | 1,795,524 | 1,795,524 | 1,795,524 | 1,795,524 | 1,795,524 | 1,795,524 | 1,795,524 |
|  | Fixed operating costs | 183,686,896 | 183,686,896 | 183,686,896 | 183,686,896 | 183,686,896 | 183,686,896 | 183,686,896 | 183,686,896 |
| Total product cost | | 4,854,296,396 | 4,854,296,396 | 4,854,296,396 | 4,854,296,396 | 4,854,296,396 | 4,854,296,396 | 4,854,296,396 | 4,854,296,396 |
| Annual depreciation | |  |  |  |  |  |  |  |  |
| General plant writedown | | |  |  |  |  |  |  |  |
|  | Depreciation charge | |  |  |  |  |  |  |  |
|  | Remaining value |  |  |  |  |  |  |  |  |
| Net revenue | | 715,778,584 | 715,778,584 | 715,778,584 | 715,778,584 | 715,778,584 | 715,778,584 | 715,778,584 | 715,778,584 |
| Losses forward | | 0 | 0 | 0 | 0 | 0 | 0 | 0 | 0 |
| Taxable income | | 715,778,584 | 715,778,584 | 715,778,584 | 715,778,584 | 715,778,584 | 715,778,584 | 715,778,584 | 715,778,584 |
| Income tax | | 150,313,503 | 150,313,503 | 150,313,503 | 150,313,503 | 150,313,503 | 150,313,503 | 150,313,503 | 150,313,503 |
| Annual cash income | | 565,465,081 | 565,465,081 | 565,465,081 | 565,465,081 | 565,465,081 | 565,465,081 | 565,465,081 | 565,465,081 |
| Discount factor | | 0.2633 | 0.2394 | 0.2176 | 0.1978 | 0.1799 | 0.1635 | 0.1486 | 0.1351 |
| Annual present value | | 148,904,629 | 135,367,845 | 123,061,677 | 111,874,252 | 101,703,865 | 92,458,059 | 84,052,781 | 76,411,619 |
| Total capital investment + interest | | |  |  |  |  |  |  |  |
| Net present worth | |  |  |  |  |  |  |  |  |

Table B.8. Discounted cash flow calculations of ORC-PEMWE (continued).

| Year | | 22 | 23 | 24 | 25 | 26 | 27 | 28 | 29 | 30 |
| --- | --- | --- | --- | --- | --- | --- | --- | --- | --- | --- |
| Fixed capital investment | | |  |  |  |  |  |  |  |  |
| Land | |  |  |  |  |  |  |  |  | -96,785,696 |
| Working fluid | |  |  |  |  |  |  |  |  | -626,128 |
| Working capital | |  |  |  |  |  |  |  |  | -241,964,240 |
| Loan payment | | 0 | 0 | 0 | 0 | 0 | 0 | 0 | 0 | 0 |
|  | Loan interest payment | 0 | 0 | 0 | 0 | 0 | 0 | 0 | 0 | 0 |
|  | Loan principal | 0 | 0 | 0 | 0 | 0 | 0 | 0 | 0 | 0 |
|  | Hydrogen sales | 42,911,497 | 42,911,497 | 42,911,497 | 42,911,497 | 42,911,497 | 42,911,497 | 42,911,497 | 42,911,497 | 42,911,497 |
|  | NG sales | 5,527,163,482 | 5,527,163,482 | 5,527,163,482 | 5,527,163,482 | 5,527,163,482 | 5,527,163,482 | 5,527,163,482 | 5,527,163,482 | 5,527,163,482 |
| Total annual sales | | 5,570,074,980 | 5,570,074,980 | 5,570,074,980 | 5,570,074,980 | 5,570,074,980 | 5,570,074,980 | 5,570,074,980 | 5,570,074,980 | 5,570,074,980 |
| Annual manufacturing cost | | |  |  |  |  |  |  |  |  |
|  | Feedstock | 4,668,813,976 | 4,668,813,976 | 4,668,813,976 | 4,668,813,976 | 4,668,813,976 | 4,668,813,976 | 4,668,813,976 | 4,668,813,976 | 4,668,813,976 |
|  | Other variable costs | 1,795,524 | 1,795,524 | 1,795,524 | 1,795,524 | 1,795,524 | 1,795,524 | 1,795,524 | 1,795,524 | 1,795,524 |
|  | Fixed operating costs | 183,686,896 | 183,686,896 | 183,686,896 | 183,686,896 | 183,686,896 | 183,686,896 | 183,686,896 | 183,686,896 | 183,686,896 |
| Total product cost | | 4,854,296,396 | 4,854,296,396 | 4,854,296,396 | 4,854,296,396 | 4,854,296,396 | 4,854,296,396 | 4,854,296,396 | 4,854,296,396 | 4,854,296,396 |
| Annual depreciation | |  |  |  |  |  |  |  |  |  |
| General plant writedown | | |  |  |  |  |  |  |  |  |
|  | Depreciation charge | |  |  |  |  |  |  |  |  |
|  | Remaining value |  |  |  |  |  |  |  |  |  |
| Net revenue | | 715,778,584 | 715,778,584 | 715,778,584 | 715,778,584 | 715,778,584 | 715,778,584 | 715,778,584 | 715,778,584 | 715,778,584 |
| Losses forward | | 0 | 0 | 0 | 0 | 0 | 0 | 0 | 0 | 0 |
| Taxable income | | 715,778,584 | 715,778,584 | 715,778,584 | 715,778,584 | 715,778,584 | 715,778,584 | 715,778,584 | 715,778,584 | 715,778,584 |
| Income tax | | 150,313,503 | 150,313,503 | 150,313,503 | 150,313,503 | 150,313,503 | 150,313,503 | 150,313,503 | 150,313,503 | 150,313,503 |
| Annual cash income | | 565,465,081 | 565,465,081 | 565,465,081 | 565,465,081 | 565,465,081 | 565,465,081 | 565,465,081 | 565,465,081 | 565,465,081 |
| Discount factor | | 0.1228 | 0.1117 | 0.1015 | 0.0923 | 0.0839 | 0.0763 | 0.0693 | 0.0630 | 0.0573 |
| Annual present value | | 69,465,108 | 63,150,099 | 57,409,181 | 52,190,164 | 47,445,604 | 43,132,367 | 39,211,243 | 35,646,584 | 32,405,986 |
| Total capital investment + interest | | |  |  |  |  |  |  |  | -19,413,269 |
| Net present worth | |  |  |  |  |  |  |  |  |  |

Table B.9. Discounted cash flow calculations of ORC-SWDE.

| Year | | -2 | -1 | 0 | 1 | 2 | 3 | 4 | 5 |
| --- | --- | --- | --- | --- | --- | --- | --- | --- | --- |
| Fixed capital investment | | 160,125,732 | 1,200,942,988 | 640,502,927 |  |  |  |  |  |
| Land | | 100,078,582 |  |  |  |  |  |  |  |
| Working fluid | |  |  | 626,128 |  |  |  |  |  |
| Working capital | |  |  | 250,196,456 |  |  |  |  |  |
| Loan payment | |  |  |  | 447,439,798 | 447,439,798 | 447,439,798 | 447,439,798 | 447,439,798 |
|  | Loan interest payment | 19,215,088 | 163,328,246 | 240,188,598 | 240,188,598 | 223,608,501 | 205,701,998 | 186,362,974 | 165,476,828 |
|  | Loan principal | 240,188,598 | 2,041,603,079 | 3,002,357,469 | 2,795,106,268 | 2,571,274,971 | 2,329,537,170 | 2,068,460,345 | 1,786,497,374 |
|  | Hydrogen sales | |  |  | 32,799,709 | 43,732,946 | 43,732,946 | 43,732,946 | 43,732,946 |
|  | NG sales | |  |  | 4,273,950,373 | 5,698,600,498 | 5,698,600,498 | 5,698,600,498 | 5,698,600,498 |
| Total annual sales | | |  |  | 4,306,750,083 | 5,742,333,444 | 5,742,333,444 | 5,742,333,444 | 5,742,333,444 |
| Annual manufacturing cost | | |  |  |  |  |  |  |  |
|  | Feedstock |  |  |  | 4,085,212,229 | 4,668,813,976 | 4,668,813,976 | 4,668,813,976 | 4,668,813,976 |
|  | Other variable costs | |  |  | 12,576,657 | 143,682,896 | 143,682,896 | 143,682,896 | 143,682,896 |
|  | Fixed operating costs | |  |  | 189,936,373 | 189,936,373 | 189,936,373 | 189,936,373 | 189,936,373 |
| Total product cost | | |  |  | 4,400,871,137 | 5,002,433,246 | 5,002,433,246 | 5,002,433,246 | 5,002,433,246 |
| Annual depreciation | | |  |  |  |  |  |  |  |
| General plant writedown | | |  |  | 14.29% | 24.49% | 17.49% | 12.49% | 8.93% |
|  | Depreciation charge | |  |  | 715,061,471 | 1,225,462,240 | 875,187,202 | 624,990,746 | 446,850,870 |
|  | Remaining value | |  |  | 4,288,867,644 | 3,063,405,404 | 2,188,218,202 | 1,563,227,455 | 1,116,376,586 |
| Net revenue | |  |  |  | -1,049,371,122 | -709,170,544 | -340,989,002 | -71,453,522 | 127,572,500 |
| Losses forward | |  |  |  |  | -1,049,371,122 | -1,758,541,665 | -2,099,530,667 | -2,170,984,189 |
| Taxable income | |  |  |  | -1,049,371,122 | -1,758,541,665 | -2,099,530,667 | -2,170,984,189 | -2,043,411,689 |
| Income tax | |  |  |  | 0 | 0 | 0 | 0 | 0 |
| Annual cash income | | |  |  | -541,560,852 | 292,460,400 | 292,460,400 | 292,460,400 | 292,460,400 |
| Discount factor | | 1.2100 | 1.1000 | 1.0000 | 0.9091 | 0.8264 | 0.7513 | 0.6830 | 0.6209 |
| Annual present value | | |  |  | -492,328,048 | 241,702,810 | 219,729,827 | 199,754,388 | 181,594,898 |
| Total capital investment + interest | | 338,097,476 | 1,500,698,357 | 1,131,514,108 |  |  |  |  |  |
| Net present worth | | |  | 0 |  |  |  |  |  |

Table B.9. Discounted cash flow calculations of ORC-SWDE (continued).

| Year | | 6 | 7 | 8 | 9 | 10 | 11 | 12 | 13 |
| --- | --- | --- | --- | --- | --- | --- | --- | --- | --- |
| Fixed capital investment | | |  |  |  |  |  |  |  |
| Land | |  |  |  |  |  |  |  |  |
| Working fluid | |  |  |  |  |  |  |  |  |
| Working capital | |  |  |  |  |  |  |  |  |
| Loan payment | | 447,439,798 | 447,439,798 | 447,439,798 | 447,439,798 | 447,439,798 | 0 | 0 | 0 |
|  | Loan interest payment | 142,919,790 | 118,558,189 | 92,247,661 | 63,832,289 | 33,143,689 | 0 | 0 | 0 |
|  | Loan principal | 1,481,977,366 | 1,153,095,757 | 797,903,619 | 414,296,110 | 0 | 0 | 0 | 0 |
|  | Hydrogen sales | 43,732,946 | 43,732,946 | 43,732,946 | 43,732,946 | 43,732,946 | 43,732,946 | 43,732,946 | 43,732,946 |
|  | NG sales | 5,698,600,498 | 5,698,600,498 | 5,698,600,498 | 5,698,600,498 | 5,698,600,498 | 5,698,600,498 | 5,698,600,498 | 5,698,600,498 |
| Total annual sales | | 5,742,333,444 | 5,742,333,444 | 5,742,333,444 | 5,742,333,444 | 5,742,333,444 | 5,742,333,444 | 5,742,333,444 | 5,742,333,444 |
| Annual manufacturing cost | | |  |  |  |  |  |  |  |
|  | Feedstock | 4,668,813,976 | 4,668,813,976 | 4,668,813,976 | 4,668,813,976 | 4,668,813,976 | 4,668,813,976 | 4,668,813,976 | 4,668,813,976 |
|  | Other variable costs | 143,682,896 | 143,682,896 | 143,682,896 | 143,682,896 | 143,682,896 | 143,682,896 | 143,682,896 | 143,682,896 |
|  | Fixed operating costs | 189,936,373 | 189,936,373 | 189,936,373 | 189,936,373 | 189,936,373 | 189,936,373 | 189,936,373 | 189,936,373 |
| Total product cost | | 5,002,433,246 | 5,002,433,246 | 5,002,433,246 | 5,002,433,246 | 5,002,433,246 | 5,002,433,246 | 5,002,433,246 | 5,002,433,246 |
| Annual depreciation | |  |  |  |  |  |  |  |  |
| General plant writedown | | 8.92% | 8.93% | 4.46% |  |  |  |  |  |
|  | Depreciation charge | 446,350,477 | 446,850,870 | 223,175,239 |  |  |  |  |  |
|  | Remaining value | 670,026,108 | 223,175,239 | 0 |  |  |  |  |  |
| Net revenue | | 150,629,931 | 174,491,139 | 424,477,299 | 676,067,909 | 706,756,509 | 739,900,198 | 739,900,198 | 739,900,198 |
| Losses forward | | -2,043,411,689 | -1,892,781,758 | -1,718,290,619 | -1,293,813,320 | -617,745,411 | 0 | 0 | 0 |
| Taxable income | | -1,892,781,758 | -1,718,290,619 | -1,293,813,320 | -617,745,411 | 89,011,098 | 739,900,198 | 739,900,198 | 739,900,198 |
| Income tax | | 0 | 0 | 0 | 0 | 18,692,331 | 155,379,042 | 155,379,042 | 155,379,042 |
| Annual cash income | | 292,460,400 | 292,460,400 | 292,460,400 | 292,460,400 | 273,768,069 | 584,521,156 | 584,521,156 | 584,521,156 |
| Discount factor | | 0.5645 | 0.5132 | 0.4665 | 0.4241 | 0.3855 | 0.3505 | 0.3186 | 0.2897 |
| Annual present value | | 165,086,271 | 150,078,428 | 136,434,935 | 124,031,759 | 105,549,442 | 204,871,099 | 186,246,454 | 169,314,958 |
| Total capital investment + interest | | |  |  |  |  |  |  |  |
| Net present worth | |  |  |  |  |  |  |  |  |

Table B.9. Discounted cash flow calculations of ORC-SWDE (continued)

| Year | | 14 | 15 | 16 | 17 | 18 | 19 | 20 | 21 |
| --- | --- | --- | --- | --- | --- | --- | --- | --- | --- |
| Fixed capital investment | | |  |  |  |  |  |  |  |
| Land | |  |  |  |  |  |  |  |  |
| Working fluid | |  |  |  |  |  |  |  |  |
| Working capital | |  |  |  |  |  |  |  |  |
| Loan payment | | 0 | 0 | 0 | 0 | 0 | 0 | 0 | 0 |
|  | Loan interest payment | 0 | 0 | 0 | 0 | 0 | 0 | 0 | 0 |
|  | Loan principal | 0 | 0 | 0 | 0 | 0 | 0 | 0 | 0 |
|  | Hydrogen sales | 43,732,946 | 43,732,946 | 43,732,946 | 43,732,946 | 43,732,946 | 43,732,946 | 43,732,946 | 43,732,946 |
|  | NG sales | 5,698,600,498 | 5,698,600,498 | 5,698,600,498 | 5,698,600,498 | 5,698,600,498 | 5,698,600,498 | 5,698,600,498 | 5,698,600,498 |
| Total annual sales | | 5,742,333,444 | 5,742,333,444 | 5,742,333,444 | 5,742,333,444 | 5,742,333,444 | 5,742,333,444 | 5,742,333,444 | 5,742,333,444 |
| Annual manufacturing cost | | |  |  |  |  |  |  |  |
|  | Feedstock | 4,668,813,976 | 4,668,813,976 | 4,668,813,976 | 4,668,813,976 | 4,668,813,976 | 4,668,813,976 | 4,668,813,976 | 4,668,813,976 |
|  | Other variable costs | 143,682,896 | 143,682,896 | 143,682,896 | 143,682,896 | 143,682,896 | 143,682,896 | 143,682,896 | 143,682,896 |
|  | Fixed operating costs | 189,936,373 | 189,936,373 | 189,936,373 | 189,936,373 | 189,936,373 | 189,936,373 | 189,936,373 | 189,936,373 |
| Total product cost | | 5,002,433,246 | 5,002,433,246 | 5,002,433,246 | 5,002,433,246 | 5,002,433,246 | 5,002,433,246 | 5,002,433,246 | 5,002,433,246 |
| Annual depreciation | |  |  |  |  |  |  |  |  |
| General plant writedown | | |  |  |  |  |  |  |  |
|  | Depreciation charge | |  |  |  |  |  |  |  |
|  | Remaining value |  |  |  |  |  |  |  |  |
| Net revenue | | 739,900,198 | 739,900,198 | 739,900,198 | 739,900,198 | 739,900,198 | 739,900,198 | 739,900,198 | 739,900,198 |
| Losses forward | | 0 | 0 | 0 | 0 | 0 | 0 | 0 | 0 |
| Taxable income | | 739,900,198 | 739,900,198 | 739,900,198 | 739,900,198 | 739,900,198 | 739,900,198 | 739,900,198 | 739,900,198 |
| Income tax | | 155,379,042 | 155,379,042 | 155,379,042 | 155,379,042 | 155,379,042 | 155,379,042 | 155,379,042 | 155,379,042 |
| Annual cash income | | 584,521,156 | 584,521,156 | 584,521,156 | 584,521,156 | 584,521,156 | 584,521,156 | 584,521,156 | 584,521,156 |
| Discount factor | | 0.2633 | 0.2394 | 0.2176 | 0.1978 | 0.1799 | 0.1635 | 0.1486 | 0.1351 |
| Annual present value | | 153,922,689 | 139,929,718 | 127,208,834 | 115,644,395 | 105,131,268 | 95,573,880 | 86,885,345 | 78,986,678 |
| Total capital investment + interest | | |  |  |  |  |  |  |  |
| Net present worth | |  |  |  |  |  |  |  |  |

Table B.9. Discounted cash flow calculations of ORC-SWDE (continued).

| Year | | 22 | 23 | 24 | 25 | 26 | 27 | 28 | 29 | 30 |
| --- | --- | --- | --- | --- | --- | --- | --- | --- | --- | --- |
| Fixed capital investment | | |  |  |  |  |  |  |  |  |
| Land | |  |  |  |  |  |  |  |  | -100,078,582 |
| Working fluid | |  |  |  |  |  |  |  |  | -626,128 |
| Working capital | |  |  |  |  |  |  |  |  | -250,196,456 |
| Loan payment | | 0 | 0 | 0 | 0 | 0 | 0 | 0 | 0 | 0 |
|  | Loan interest payment | 0 | 0 | 0 | 0 | 0 | 0 | 0 | 0 | 0 |
|  | Loan principal | 0 | 0 | 0 | 0 | 0 | 0 | 0 | 0 | 0 |
|  | Hydrogen sales | 43,732,946 | 43,732,946 | 43,732,946 | 43,732,946 | 43,732,946 | 43,732,946 | 43,732,946 | 43,732,946 | 43,732,946 |
|  | NG sales | 5,698,600,498 | 5,698,600,498 | 5,698,600,498 | 5,698,600,498 | 5,698,600,498 | 5,698,600,498 | 5,698,600,498 | 5,698,600,498 | 5,698,600,498 |
| Total annual sales | | 5,742,333,444 | 5,742,333,444 | 5,742,333,444 | 5,742,333,444 | 5,742,333,444 | 5,742,333,444 | 5,742,333,444 | 5,742,333,444 | 5,742,333,444 |
| Annual manufacturing cost | | |  |  |  |  |  |  |  |  |
|  | Feedstock | 4,668,813,976 | 4,668,813,976 | 4,668,813,976 | 4,668,813,976 | 4,668,813,976 | 4,668,813,976 | 4,668,813,976 | 4,668,813,976 | 4,668,813,976 |
|  | Other variable costs | 143,682,896 | 143,682,896 | 143,682,896 | 143,682,896 | 143,682,896 | 143,682,896 | 143,682,896 | 143,682,896 | 143,682,896 |
|  | Fixed operating costs | 189,936,373 | 189,936,373 | 189,936,373 | 189,936,373 | 189,936,373 | 189,936,373 | 189,936,373 | 189,936,373 | 189,936,373 |
| Total product cost | | 5,002,433,246 | 5,002,433,246 | 5,002,433,246 | 5,002,433,246 | 5,002,433,246 | 5,002,433,246 | 5,002,433,246 | 5,002,433,246 | 5,002,433,246 |
| Annual depreciation | |  |  |  |  |  |  |  |  |  |
| General plant writedown | | |  |  |  |  |  |  |  |  |
|  | Depreciation charge | |  |  |  |  |  |  |  |  |
|  | Remaining value |  |  |  |  |  |  |  |  |  |
| Net revenue | | 739,900,198 | 739,900,198 | 739,900,198 | 739,900,198 | 739,900,198 | 739,900,198 | 739,900,198 | 739,900,198 | 739,900,198 |
| Losses forward | | 0 | 0 | 0 | 0 | 0 | 0 | 0 | 0 | 0 |
| Taxable income | | 739,900,198 | 739,900,198 | 739,900,198 | 739,900,198 | 739,900,198 | 739,900,198 | 739,900,198 | 739,900,198 | 739,900,198 |
| Income tax | | 155,379,042 | 155,379,042 | 155,379,042 | 155,379,042 | 155,379,042 | 155,379,042 | 155,379,042 | 155,379,042 | 155,379,042 |
| Annual cash income | | 584,521,156 | 584,521,156 | 584,521,156 | 584,521,156 | 584,521,156 | 584,521,156 | 584,521,156 | 584,521,156 | 584,521,156 |
| Discount factor | | 0.1228 | 0.1117 | 0.1015 | 0.0923 | 0.0839 | 0.0763 | 0.0693 | 0.0630 | 0.0573 |
| Annual present value | | 71,806,071 | 65,278,246 | 59,343,860 | 53,948,964 | 49,044,512 | 44,585,920 | 40,532,655 | 36,847,868 | 33,498,062 |
| Total capital investment + interest | | |  |  |  |  |  |  |  | -20,073,756 |
| Net present worth | |  |  |  |  |  |  |  |  |  |

**C. Life cycle assessment (LCA)**

The SWDE material inventory was estimated by applying the replacement frequency ratio between PEMWE and SWDE. Replacement intervals of 40,000 h for PEMWE and 500 h for SWDE were used, corresponding to an 80 times difference in replacement frequency. The resulting inventory was subsequently normalized to the functional unit.

Table C.1. List of input/output materials.

| ORV | | | | | |  | ORC | | | | |
| --- | --- | --- | --- | --- | --- | --- | --- | --- | --- | --- | --- |
| Input | | | | Output | |  | Input | | | Output | |
| LNG ^a^ | 1.36E+00 | | | NG ^c^ | 1.00E+00 |  | LNG ^a^ | 1.36E+00 | NG ^c^ | | 1.00E+00 |
| Electricity ^b^ | 6.41E-03 | | |  |  |  |  |  | R170 ^d^ | | 1.22E-06 |
|  |  | | |  |  |  |  |  | R290 ^d^ | | 1.56E-06 |
|  |  | | |  |  |  |  |  | Electricity ^b^ | | 2.07E-02 |
| ORC-PEMWE | | | | | |  | ORC-SWDE | | | | |
| Input | | | | Output | |  | Input | | | Output | |
| LNG ^a^ | | 1.36E+00 | | NG | 1.00E+00 |  | LNG ^a^ | 1.36E+00 | NG | | 1.00E+00 |
|  | |  | | R170 ^d^ | 1.22E-06 |  |  |  | R170 ^d^ | | 1.22E-06 |
|  | |  | | R290 ^d^ | 1.56E-06 |  |  |  | R290 ^d^ | | 1.56E-06 |
|  | |  | | Desalina-tion ^e^ | 3.26E-03 |  |  |  | SWDE ^g^ | | 2.98E-02 |
|  | |  | | DI system | 3.26E-03 |  |  |  | Hydrogen | | 3.72E-04 |
|  | | |  | PEMWE ^f^ | 3.63E-04 |  |  |  |  | |  |
|  | | |  | Hydrogen | 3.63E-04 |  |  |  |  | |  |
| ^a^ m^3^.  ^b^ kWh.  ^c^ kg.  ^d^ kg. A replacement rate of 2% was assumed.  ^e^ kg of produced DI water.  ^f^ Taken from Krishnan et al. [10]  ^g^ Determined by comparison with the lifetime of PEMWE. The lifetime was taken from Liu et al. [5] | | | | | | | | | | | |

Table C.2. Background datasets used in the life cycle inventory.

| Materials | Name of data set | Database |
| --- | --- | --- |
| LNG | market for natural gas, liquefied [GLO] | ecoinvent 3.12 |
| Electricity | electricity, medium voltage [GLO] | ecoinvent 3.12 |
| R170 | market for ethane [RoW] | ecoinvent 3.12 |
| R290 | market for propane [RoW] | ecoinvent 3.12 |
| RO membrane | tap water production, seawater reverse osmosis, ultrafiltration pretreatment, enhance module, two stages [GLO] | ecoinvent 3.12 |
| Hydrogen | market for hydrogen, gaseous, low pressure [GLO] | ecoinvent 3.12 |
| PEMWE unit |  |  |
| DI system | water production, deionised [RoW] | ecoinvent 3.12 |
| TFE | market for tetrafluoroethylene [GLO] | ecoinvent 3.12 |
| Carbon black | market for carbon black [GLO] | ecoinvent 3.12 |
| Platinum | market for platinum | ecoinvent 3.12 |
| DI water | market for water, deionised [RoW] | ecoinvent 3.12 |
| Methanol | market for methanol [RoW] | ecoinvent 3.12 |
| Rhodium | market for rhodium | ecoinvent 3.12 |
| Titanium | market for titanium | ecoinvent 3.12 |
| Gold | market for gold | ecoinvent 3.12 |
| Glass fiber | market for glass fibre reinforced plastic, polyamide, injection moulded | ecoinvent 3.12 |
| Steel | market for steel, chromium steel 18/8 | ecoinvent 3.12 |
| Aluminium alloy | market for aluminium alloy, metal matrix composite | ecoinvent 3.12 |

Table C.3. Life cycle environmental impacts of the evaluated configurations per functional unit of 1 kg NG.

|  | Unit | ORV | ORC | ORC-PEMWE | ORC-SWDE |
| --- | --- | --- | --- | --- | --- |
| Acidification | mol H^+^-Eq | 3.60E-03 | 3.50E-03 | 3.58E-03 | 4.12E-03 |
| Climate change | kg CO_2_-Eq | 7.85E-01 | 7.67E-01 | 7.77E-01 | 7.89E-01 |
| Ecotoxicity: freshwater | CTUe | 5.01E+00 | 4.96E+00 | 5.00E+00 | 5.31E+00 |
| Energy resources: non-renewable | MJ, net calorific value | 5.81E+01 | 5.78E+01 | 5.80E+01 | 5.81E+01 |
| Eutrophication: freshwater | kg P-Eq | 5.57E-05 | 4.70E-05 | 5.36E-05 | 7.02E-05 |
| Eutrophication: terrestrial | kg N-Eq | 1.07E-02 | 1.05E-02 | 1.06E-02 | 1.14E-02 |
| Ionising radiation:human health | kBq U235-Eq | 8.25E-03 | 5.79E-03 | 7.65E-03 | 8.12E-03 |
| Land use | dimensionless | 5.67E-01 | 5.31E-01 | 5.58E-01 | 6.50E-01 |
| Ozone depletion | kg CFC-11-Eq | 1.08E-07 | 1.08E-07 | 1.08E-07 | 1.11E-07 |
| Particulate matter formation | disease incidence | 1.13E-08 | 1.05E-08 | 1.11E-08 | 1.28E-08 |
| Photochemical oxidant formation: human health | kg NMVOC-Eq | 5.66E-03 | 5.61E-03 | 5.65E-03 | 5.83E-03 |
| Water use | m^3^ world Eq deprived | 4.61E-02 | 4.16E-02 | 4.46E-02 | 4.70E-02 |

Table C.4. LCA data for sensitivity analysis.

| Name of data set | Database | Climate change |
| --- | --- | --- |
| market for electricity, medium voltage [CN-CCG] | ecoinvent 3.12 | 0.8505 ^a^ |
| market for electricity, medium voltage [KR] | ecoinvent 3.12 | 0.6700 ^a^ |
| market for electricity, medium voltage [JP] | ecoinvent 3.12 | 0.6417 ^a^ |
| market for electricity, medium voltage [SE] | ecoinvent 3.12 | 0.0306 ^a^ |
| market for electricity, medium voltage [IN-Eastern grid] | ecoinvent 3.12 | 1.5525 ^a^ |
| electricity production, oil [RoW] | ecoinvent 3.12 | 1.0110 ^a^ |
| electricity production, coal, aluminium industry [RoW] | ecoinvent 3.12 | 1.1667 ^a^ |
| electricity production, nuclear, boiling water reactor [RoW] | ecoinvent 3.12 | 0.0077 ^a^ |
| electricity production, hydro, pumped storage [RoW] | ecoinvent 3.12 | 0.9608 ^a^ |
| electricity production, lignite [RoW] | ecoinvent 3.12 | 1.3075 ^a^ |
| electricity production, hard coal [RoW] | ecoinvent 3.12 | 1.0864 ^a^ |
| market for hydrogen, gaseous, low pressure [CN] | ecoinvent 3.12 | 18.1668 ^b^ |
| market for hydrogen, gaseous, low pressure [RER] | ecoinvent 3.12 | 9.5478 ^b^ |
| market for hydrogen, gaseous, low pressure [RoW] | ecoinvent 3.12 | 9.5341 ^b^ |
| market for hydrogen, gaseous, low pressure [US] | ecoinvent 3.12 | 9.9781 ^b^ |
| ^a^ kg CO_2_-Eq/kWh |  |  |
| ^b^ kg CO_2_-Eq/kg |  |  |

The sensitivity of the ORC–PEMWE result to the substituted hydrogen dataset was evaluated using regional market datasets for gaseous hydrogen. The functional unit, system boundary, impact assessment method, and 1:1 substitution ratio was maintained, while only the geographic scope of the substituted hydrogen dataset were changed.

The calculated GWP of ORC–PEMWE ranged from 0.7457 to 0.7772 kg CO_2_-eq/kg NG. A hydrogen market dataset with a greater upstream greenhouse gas burden resulted in a larger substitution credit and a lower net GWP for the integrated system. Although the magnitude of the environmental benefit varied according to the regional hydrogen dataset, all evaluated results remained below the ORV result of 0.7851 kg CO_2_-eq/kg NG. Therefore, the principal environmental conclusion was not affected by the selection of the substituted hydrogen dataset.

Figure C.1. Sensitivity of the GWP of the ORC–PEMWE configuration to the geographic scope of the avoided gaseous hydrogen market dataset used in the system expansion analysis.
**D. Thermodynamic performance of the ORC system**

The thermodynamic performance of the parallel ORC-integrated LNG regasification system was evaluated at the terminal-level LNG throughput of 2,160,000 kg/h. The turbine power outputs are presented as positive values, although Aspen Plus reports generated shaft work using a negative sign convention. The heat exchanger duties are also reported as absolute values.

The total turbine power output was calculated as the sum of power outputs from the R170 and R290 turbines. The ORC net power output was determined by subtracting the working fluid pump power consumption from the total turbine power output. The terminal net power output was calculated by additionally subtracting the power consumption of the LNG and seawater pumps. Therefore, the terminal net power output represents the electricity available for export or downstream hydrogen production.

The net thermal efficiency and specific net power output were calculated using Eqs. (D.1) and (D.2), respectively. In Eq. (D.1) $\eta_{net}$ represents the net thermal efficiency of the ORC-integrated LNG regasification system, $W_{net, terminal}$ is the terminal net power output after accounting for the power consumption of the working fluid, LNG, and seawater pumps, and $Q_{H-3}$ and $Q_{H-5}$ denote the heat duties supplied from seawater to the R170 and R290 ORC loops, respectively. In Eq. (D.2), Specific net power output(SNPO) represents the specific net power output, and $\dot{m}_{LNG}$ is the LNG mass flow rate.

$\eta_{net}=\frac{W_{net, terminal}}{\left| Q_{H-3} \right|+\left| Q_{H-5} \right|}\times100$	(D.1)

$SNPO=\frac{W_{net, terminal}}{\dot{m}_{LNG}}$	(D.2)

Table D.1. Terminal-level thermodynamic performance of the parallel ORC-integrated LNG regasification system.

| Parameter indicator | Value | |
| --- | --- | --- |
| LNG mass flow ^a^ | | 2,160,000 |
| R170 turbine power output ^b^ | | 36.56 |
| R290 turbine power output ^b^ | | 24.11 |
| **Total turbine power output ^b^** | | **60.67** |
| R170 working fluid pump consumption ^b^ | | 1.34 |
| R290 working fluid pump consumption ^b^ | | 0.50 |
| **Total working fluid pump consumption ^b^** | | **1.84** |
| **ORC net power output ^b^** | | **58.83** |
| LNG pump consumption ^b^ | | 12.52 |
| Seawater pump consumption ^b^ | | 1.61 |
| **Total pump consumption ^b^** | | **15.97** |
| **Terminal net power output ^b^** | | **44.70** |
| H-2 heat duty, R170 condenser ^b^ | | 138.45 |
| H-3 heat duty, R170 evaporator ^b^ | | 173.67 |
| H-4 heat duty, R290 condenser ^b^ | | 156.52 |
| H-5 heat duty, R290 evaporator ^b^ | | 180.13 |
| Net thermal efficiency ^c^ | | 12.63 |
| Specific net power output ^d^ | | 74.50 |
| ^a^ kg/h |  | |
| ^b^ MW |  | |
| ^c^ % |  | |
| ^d^ kJ/kg-LNG |  | |

**References**

[1] P. J. Linstrom and W. G. Mallard, Eds., *NIST Chemistry WebBook, NIST Standard Reference Database Number 69*, National Institute of Standards and Technology, Gaithersburg, MD, 2025. doi:10.18434/T4D303.

[2] World Bank, *The Role of Desalination in an Increasingly Water-Scarce World*, Water Global Practice Technical Paper, World Bank, Washington, DC, 2019, Report No. 135312.

[3] M. Hubert, D. Peterson, E. Miller, J. Vickers, R. Mow, and C. Howe, *Clean Hydrogen Production Cost Scenarios with PEM Electrolyzer Technology*, DOE Hydrogen Program Record 24005, U.S. Department of Energy, 2024.

[4] R. Davis, N. Grundl, L. Tao, M. J. Biddy, E. C. D. Tan, G. T. Beckham, D. Humbird, D. N. Thompson, and M. S. Roni, *Process Design and Economics for the Conversion of Lignocellulosic Biomass to Hydrocarbon Fuels and Coproducts: 2018 Biochemical Design Case Update: Biochemical Deconstruction and Conversion of Biomass to Fuels and Products via Integrated Biorefinery Pathways*, National Renewable Energy Laboratory, Golden, CO, NREL/TP-5100-71949, 2018. doi:10.2172/1483234.

[5] T. Liu, Z. Zhao, W. Tang, Y. Chen, C. Lan, L. Zhu, W. Jiang, Y. Wu, Y. Wang, Z. Yang, D. Yang, Q. Wang, L. Luo, T. Liu, and H. Xie, “In-Situ Direct Seawater Electrolysis Using Floating Platform in Ocean with Uncontrollable Wave Motion,” *Nature Communications* 2024, 15, 5305. doi:10.1038/s41467-024-49639-6.

[6] U.S. Energy Information Administration, *Henry Hub Natural Gas Spot Price*, accessed July 2026.

[7] Trading Economics, *Ethane*, accessed July 2026.

[8] Trading Economics, *Propane*, accessed July 2026.

[9] M. P. Bailey, “Chemical Engineering Plant Cost Index (CEPCI),” *Chemical Engineering* 2014, *121*, no. 2, 68–69.

[10] S. Krishnan, B. Corona, G. J. Kramer, H. M. Junginger, and V. Koning, “Prospective LCA of Alkaline and PEM Electrolyser Systems,” *International Journal of Hydrogen Energy* 2024, *55*, 26–41. doi:10.1016/j.ijhydene.2023.10.192.
